# *Neochloris oleoabundans* is worth its salt: Transcriptomic analysis under salt and nitrogen stress

**DOI:** 10.1371/journal.pone.0194834

**Published:** 2018-04-13

**Authors:** Lenny de Jaeger, Benoit M. Carreres, Jan Springer, Peter J. Schaap, Gerrit Eggink, Vitor A. P. Martins Dos Santos, Rene H. Wijffels, Dirk E. Martens

**Affiliations:** 1 Bioprocess Engineering and AlgaePARC, Wageningen University & Research, Wageningen, The Netherlands; 2 Laboratory of Systems and Synthetic Biology, Wageningen University & Research, Wageningen, The Netherlands; 3 Food and Biobased Research and AlgaePARC, Wageningen University & Research, Wageningen, The Netherlands; 4 LifeGlimmer GmbH, Berlin, Germany; 5 Nord University, Bodø, Norway; National Autonomous University of Mexico, MEXICO

## Abstract

*Neochloris oleoabundans* is an oleaginous microalgal species that can be cultivated in fresh water as well as salt water. Using salt water gives the opportunity to reduce production costs and the fresh water footprint for large scale cultivation. Production of triacylglycerols (TAG) usually includes a biomass growth phase in nitrogen-replete conditions followed by a TAG accumulation phase under nitrogen-deplete conditions. This is the first report that provides insight in the saline resistance mechanism of a fresh water oleaginous microalgae. To better understand the osmoregulatory mechanism of *N*. *oleoabundans* during growth and TAG accumulating conditions, the transcriptome was sequenced under four different conditions: fresh water nitrogen-replete and -deplete conditions, and salt water (525 mM dissolved salts, 448mM extra NaCl) nitrogen-replete and -deplete conditions. In this study, several pathways are identified to be responsible for salt water adaptation of *N*. *oleoabundans* under both nitrogen-replete and -deplete conditions. Proline and the ascorbate-glutathione cycle seem to be of importance for successful osmoregulation in *N*. *oleoabundans*. Genes involved in Proline biosynthesis were found to be upregulated in salt water. This was supported by Nuclear magnetic resonance (NMR) spectroscopy, which indicated an increase in proline content in the salt water nitrogen-replete condition. Additionally, the lipid accumulation pathway was studied to gain insight in the gene regulation in the first 24 hours after nitrogen was depleted. Oil accumulation is increased under nitrogen-deplete conditions in a comparable way in both fresh and salt water. The mechanism behind the biosynthesis of compatible osmolytes can be used to improve *N*. *oleoabundans* and other industrially relevant microalgal strains to create a more robust and sustainable production platform for microalgae derived products in the future.

## 1 Introduction

Sustainable and renewable production of energy and food for an increasing world population is an enduring challenge in present-day research. This challenge must be addressed with urgency, because of the world’s dependence on limited fossil fuels and the increase in living standards of emerging economies. Renewable energy platforms based on oleaginous agricultural crops such as rapeseed, palm oil, corn, and soybean are being studied. Although these crops are considered renewable and bio-based, they increase the competition for food, fresh water, the amount of available arable land and result in deforestation to create plantations [1–4]. Ideally, we would use land that is not suitable for traditional agriculture such as salt contaminated land or very dry areas like deserts for this purpose. A promising alternative feedstock compared to traditional crops for the production of oil are microalgae [5,6]. Microalgae can produce high amounts of neutral lipids, triacylglycerol (TAG), when exposed to unfavorable growth conditions such as nitrogen depletion. TAG can be easily converted in biodiesels by methylation, which results in a pure and clean fuel that can replace petroleum-derived fuels [5]. The TAG molecules can also be used directly in the food and feed industry as a sustainable vegetable oil replacement. For an acceptable sustainable production process with a reduced fresh water footprint the use of marine or salt tolerant microalgal species is essential.

Most organisms are not able to cope with a shift in osmotic pressure when the environment is changed from fresh water to salt water and their growth will be compromised. Some organisms are able to adapt to such changes. Plants have developed different strategies to deal with osmotic stress. In addition to the strategies that involve structural traits such as waxes and adaptation of flowering time to the right conditions and moment, plants can also regulate their osmotic homeostasis by actively excluding salts from the cell to maintain water absorption [7–10]. Another strategy involves the accumulation of certain compatible organic osmolytes. A few examples of these compatible osmolytes are proline and glycine betaine [11], cyclic polyols such as D-pinitol [12], and sugars such as sucrose hexoses and sugar alcohols [7,13].

Prokaryotic microalgae (cyanobacterias) are known to accumulate sucrose or α-glocosylglycerol under salt stress conditions [14,15]. In eukaryotic microalgae, there are some strategies that can be found to overcome salt stress[16–20]. Some species of microalgae are known to be able to survive low levels of saline environments. Known mechanisms for salt tolerance are glycerol production [21], sucrose production [22], and amino acid accumulation [7,23].

The oleaginous salt tolerant microalgae *Neochloris oleoabundans* is a very interesting candidate for lipid production [22,24,25]. In nitrogen-deplete conditions, *N*. *oleoabundans* can accumulate TAG at up to 44% of its dry weight resulting in a maximal productivity of 164 mg L^-1^ day^-1^ [26,27]. The harsh desert conditions from which this oleaginous microalgae was isolated [28], forced *N*. *oleoabundans* to be a highly flexible species to deal with the daily salt, drought and temperature stresses during the hot days and cold nights. These properties, combined with the high growth rate of *N*. *oleoabundans* (μ 2.2 D^-1^) [25], and its resistance to highly alkaline conditions (up to pH 10) [24], which enhances the CO_2_ transfer and reduces risk of contamination, makes it a very interesting candidate for sustainable oil production. The aim of this study is to identify the mechanisms used by the green microalgae *N*. *oleoabundans* to cope with saline conditions under growth (nitrogen-replete) and TAG accumulating (nitrogen-deplete) conditions based on a transcriptomic approach. To obtain insight in the pathways and metabolic reactions involved in salt resistance and lipid accumulation differential gene expression was studied between four conditions being: fresh water nitrogen-replete and -deplete, and salt water nitrogen-replete and -deplete (Panel A in S1 Fig).

To our knowledge, *N*. *oleoabundans* is the first fresh water microalgae, studied on transcriptomic and metabolite level, which is able to alleviate the osmotic stress under salt water conditions. We will discuss and compare the different pathways that are involved in the saline and nitrogen stress response. These findings can be used to get a better understanding of these processes and to define targets for new strategies to enhance microalgal strains to increase lipid productivity in the future.

## 2 Material and methods

### 2.1 Strain, medium and pre-culture

*Neochloris oleoabundans* UTEX 1185 (University of Texas, Austin, USA) pre-cultures were maintained in 100 mL filter sterilized fresh or salt water medium in 250 mL Erlenmeyer shake flasks. The fresh water medium consisted of: KNO_3_ 50.5 mM; Na_2_SO_4_ 4.6 mM; HEPES 100 mM; MgSO_4_.7H_2_O 1 mM; CaCl_2_.2H_2_O 0.5 mM; K_2_HPO_4_ 4.1 mM; NaHCO_3_ 10 mM; NaFeEDTA 0.14 mM; Na_2_.EDTA.2H_2_O 0.4 mM; MnCl_2._4H_2_O 96 μM; ZnSO_4_.7H_2_O 21 μM; CoCl_2_.6H_2_O 6 μM; CuSO_4_.5H_2_O 6.6 μM; Na_2_MoO_4_.2H_2_O 0.5 μM; biotin 0.2 μM; vitamin B1 7.4 μM; vitamin B12 0.2 μM. The same medium was used for salt water with the following modifications: NaCl 448 mM; MgSO_4_.7H_2_O 5 mM; CaCl_2_.2H_2_O 2.4 mM. The pH for both media was set to pH 7.5 using NaOH and the medium was filter sterilized (0.2 μm) prior to use. In nitrogen-deplete conditions were applied the KNO_3_ was omitted and replaced by an equimolar amount of KCl. In the shake flasks, pH was buffered using HEPES, while in the bioreactor HEPES was omitted and the pH was controlled by CO_2_ addition.

### 2.2 Reactor design

All experiments were performed in flat-panel, Algaemist airlift-loop photobioreactors [29]. The reactors, with a working volume of 0.38 L, were kept at 25°C and pH controlled using on demand CO_2_ (pH 7.5). Light was supplied continuously and the incident light intensity was adjusted to maintain constant average light intensity (80 μmol m^-2^ s^-1^). The cultures were inoculated at a biomass concentration of 0.15 g L^-1^ in either fresh or salt water medium. When the biomass concentration reached approximately 4.5 g L^-1^, the cultures were harvested and washed with either salt or fresh water medium containing nitrogen or no nitrogen. The cells were cultivated for 24 hours before sampling. Samples for dry weight, total fatty acid, TAG, starch, Nuclear magnetic resonance (NMR) analysis, and RNA extraction were taken. All reactors were run in duplicate, resulting in 8 individual samples for four different conditions.

### 2.3 Determination of dry weight concentration

Dry weight concentrations were determined on biological replicates. Around 1.5 mg of biomass was filtered through pre-dried (100°C overnight) and pre-weight Whatman glass fiber filter paper (GF/F; Whatman International Ltd, Maidstone, UK). The filter was washed with 50 mL of filtered demineralized water supplemented with an equimolar concentration of NH_4_HCO_2_ to prevent osmotic shock and subsequently dried overnight at 100°C before weighing.

### 2.4 Starch analysis

The starch content was analyzed using the Total Starch assay (Megazyme International, Wicklow, Ireland) following the protocol described previously [30]. The method is based on enzymatic degradation of starch to glucose monomers by α-amylase and amyloglucosidase enzymes and measuring glucose monomers in a spectrophotometric-based assay for quantification against a D-glucose calibration control series at a wavelength of 510 nm.

### 2.5 Total fatty acid analysis

Total fatty acid (TFA) extraction and quantification were executed as described by Breuer *et al*. [31] with the following adjustments. Around 5 mg of pellet was transferred to bead beating tubes (Lysing Matrix E; MP Biomedicals, Santa Ana, CA, USA) and lyophilized overnight. Freeze-dried cells were disrupted by a 30-min bead beating step in the presence of a chloroform-methanol mixture (1:1.25) to extract the lipids from the biomass. Tripentadecanoin (T4257; Sigma-Aldrich, St Louis, MO, USA) internal standard was added to the extraction mixture to enable fatty acid quantification. For TFA analysis, samples were directly methylated (see below). For TAG analysis, directly after the TFA extraction, the chloroform methanol mixture was evaporated under N_2_ gas and the TFA fraction was dissolved in 1 mL hexane and separated based on polarity using a Sep-Pak Vac silica cartridge (6 cc, 1,000 mg; Waters, Milford, MA, USA) prewashed with 6 mL of hexane. The neutral TAG fraction was eluted with 10 mL of hexane-diethyl ether (87:13% v/v). The polar lipid fraction containing the glycolipids and phospholipids remained in the silica cartridge. Methylation of the fatty acids to fatty acid methyl esters (FAMEs) and the quantification of the FAMEs were performed as described by Breuer [31].

### 2.6 Bodipy staining

The presence of neutral lipid bodies in *N*. *oleoabundans* was measured by staining the cells with the fluorescent dye BODIPY 505/515 (4,4-difluoro-1,3,5,7-tetramethyl-4-bora-3a,4a-diazasindacene; Invitrogen Molecular Probes, Carlsbad, CA). An aliquot of 200 μL of cells was incubated for 10 minutes with 4μL of a 40 μM BODIPY stock solution (in 0.2% (v/v) DMSO) and subsequently studied using a confocal laser scanning microscope (LSM510; Carl Zeiss, Jena, Germany), using a 488 nm Argon Laser [32]. To increase the visualization, a color filter was applied to visualize the BODIPY fluorescence as yellow signal.

### 2.7 NMR analysis

NMR analysis was performed according to the protocol described by Kim et al. [33] with the following modifications. Freeze dried microalgal biomass (20 mg) was dissolved in 0.5 ml of 50% deuterated methanol in buffer (90 mM KH_2_PO_4_ in D_2_O) containing 0.05% trimethyl silyl propionic acid sodium salt (TMSP, w/v) as internal standard. To effectively extract the metabolites, the cell suspension was vortexed and ultrasonicated for 10 min and centrifuged at 17,000xg for 5 min. From the supernatant, 300 μl was used to perform NMR analysis. The NMR analysis and data analysis was carried out as described by Kim et al. [34]. The NMR plots can be found in S3 Fig.

### 2.8 RNA extraction

Samples for RNA isolation were immediately processed after sampling and kept on ice. Cells were collected by centrifugation at 4.000xg at 0°C for three minutes and immediately frozen in liquid nitrogen before storage at -80°C until further extraction. Cells were disrupted by grinding the pellet using mortar and pestle and liquid nitrogen. A 5-ml volume of heated (65°C) phenol-chloroform and 5 ml of extraction buffer (10 mM EDTA, 1% sodium dodecyl sulphate (SDS), 2% 2-mercaptoethaol and 200 mM sodium acetate, pH 5) was added to the ground biomass. The RNA was precipitated by addition of 1/3 volume 8M lithium chloride (LiCl) enriched with 1% 2-mercaptoethanol. The RNA pellet was washed with 2M LiCl and twice with 70% ethanol. After evaporation of the last residues of ethanol, the pellet was resuspended in RNase free H_2_O and sequencing and quality control was outsourced to BaseClear BV (Leiden, The Netherlands).

### 2.9 RNA sequencing and transcriptome assembly

RNA-seq library preparation and deep sequencing on Illumina HiSeq2500 instruments were carried out at BaseClear, (Leiden, The Netherlands). The Illumina TruSeq RNA sample preparation protocol was used to prepare libraries with a median fragment insert size of 230 bp. For all samples, 51 nt paired-end sequencing runs were carried out, data was delivered in Illumina format 1.8 and filtered for reads that did not pass the Illumina chastity filter, reads that aligned to the phiX control genome and reads that contained Illumina TruSeq adapters. The sequence data can be found at EBI ArrayExpress with accession number E-MTAB-3746.

To maximize the diversity and completeness of the *N*. *oleoabundans de novo* assembled transcripts, the data from 16 transcriptomes were combined yielding a total of 496,158,724 high quality reads and assembled with IDBA-UD v1.1.0 [35]. QUAST v2.3 [36] was used to estimate the quality of the assembly. The average number of read pairs per experiments was approximately 18 million, the GC content was 59%. Coding sequences (CDS) were extracted using QUAST and translated into protein sequences for functional annotation.

### 2.10 Transcriptome annotation

The protein sequences were annotated by aligning them against different databases by using DELTA-BLAST 2.2.29+ (default parameters, E-value < 0.001) [37] and by using InterproScan 5 (default parameters) for domain search analysis. Blast2GO V.2.7 [38] was used as the central tool to combine both analysis methods to assign GO terms to the protein sequences and to retrieve EC numbers. For DELTA-BLAST the following databases were sequentially used: SwissProt, Chlorophyceae branch from SwissProt, Viridiplantae and Cyanobacteria branch from NCBI, Uniprot (SwissProt + Trembl) filtered for proteins with an annotated enzymatic reaction.

Three tools were used to predict the subcellular location of the predicted enzymes: TargetP [39], PredAlgo [40] and WoLF PSORT [41]. Four common cellular locations were identified: chloroplast, mitochondrion, secretory pathway, and cytoplasmic. In the case of WoLF-PSORT, the localizations defined as extracellular or plasma membrane were considered as secretory pathways.

### 2.11 Expression analysis

Read abundance estimations were done using the RSEM script from TrinityRNAseq [42] with the default settings. Reads from each experimental condition were mapped onto the set of coding sequences generated with QUAST from the assembled transcriptome.

Data was normalized taking into account the library sizes using Trimmed Mean of M-values [43]. They were further normalized by the CDS length to compute Fragments Per Kilo base of exon per Million fragments mapped (FPKM) using TrinityRNAseq TMM normalization script. CDS with FPKM values lower than 10 in all conditions were discarded and resulted in a reduction of ~13%. Finally, FPKM values corresponding to CDS annotated to the same enzyme or transporter were added up to provide a single expression value for the reactions in the model.

## 3 Results and discussion

### 3.1 Biomass composition

**Fig 1:**
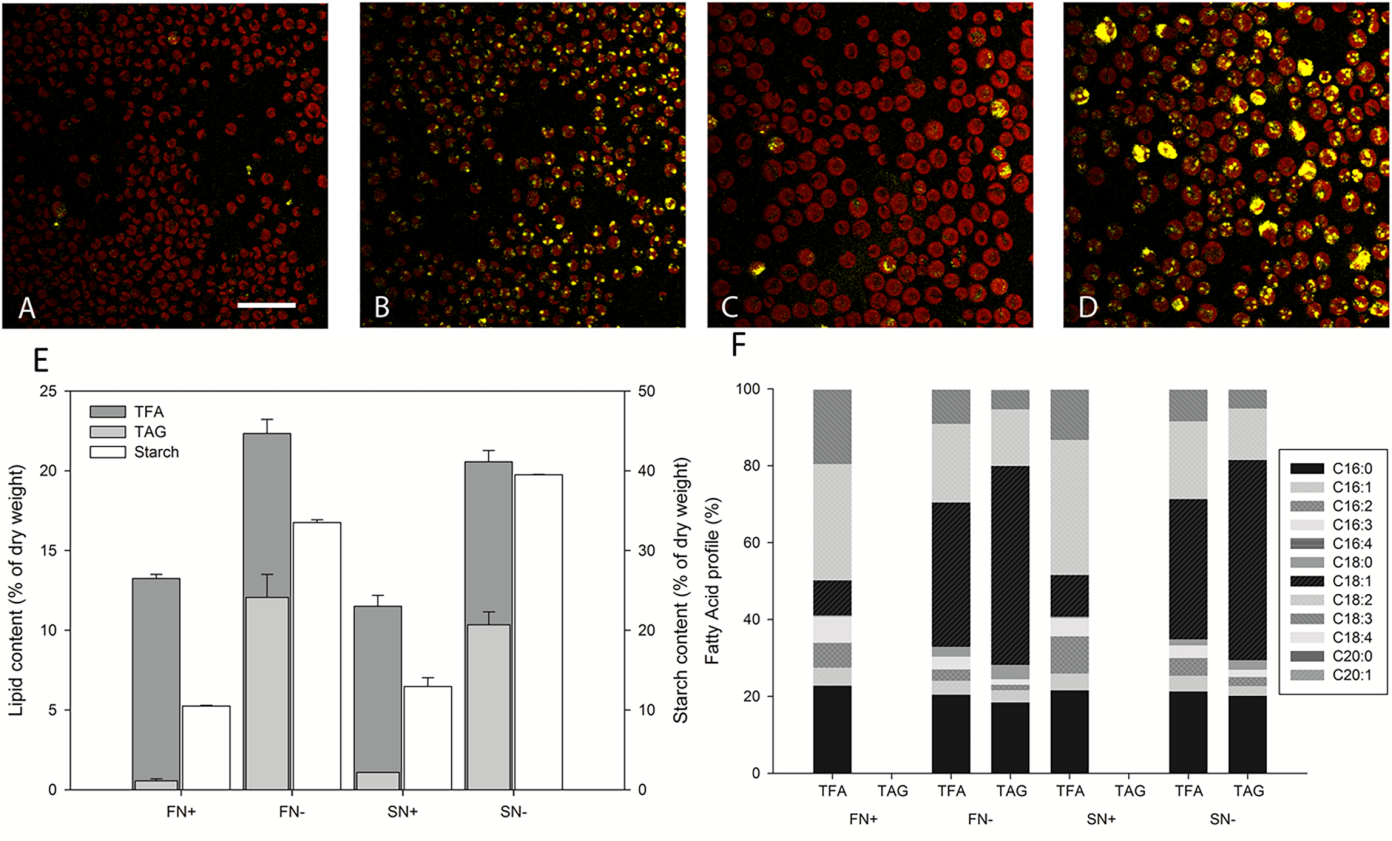
Biomass composition of *N*. *oleoabundans* in the four tested conditions. Upper panel: Confocal laser scanning microscope images of *N*. *oleoabundans* under four different cultivation conditions. (A) Nitrogen-replete fresh water (FN+). (B) Nitrogen-deplete fresh water conditions (FN-). (C) Nitrogen-replete salt water conditions (SN+). (D) Nitrogen-deplete salt water conditions (SN-). Chlorophyll autofluorescence is shown in red and the BODIPY stain is shown in yellow. The bar represents 20 μm. Lower panel: (E) Lipid and starch content. TFA (dark grey), TAG (light grey), and starch (white) 24 hours after medium replacement. The height of the bars represents the average of the two independent measurements. Error bars represent distance of the sample values to the average value. TAG, TFA and starch are given as a percentage of total dry weight. (F) Fatty acid profile under the different conditions expressed as percentage of TFA.

*N*. *oleoabundans* was cultivated under four different conditions to assess the difference in gene expression during growth (nitrogen-replete) and TAG accumulation (nitrogen-deplete) in fresh water (A and B) and salt water (C and D), and the difference in gene expression for fresh and salt water at nitrogen-replete conditions (growth) (A and C) and nitrogen-deplete conditions (TAG accumulation) (B and D) (Panel A in S1 Fig). Samples were taken at a time point when Triacylglycerol (TAG) accumulation was induced but cells were not so stressed that apoptotic gene expression dominated the transcriptional profile (Panel B in S1 Fig). In Fig (1A–1D), oil body formation in the *N*. *oleoabundans* cells is shown in the four different growth conditions. The size of the cells grown in fresh water is much smaller with a diameter of approximately 4 μm, compared to the size of cells grown in salt water with a diameter of approximately 8 μm. The red fluorescence represents the autofluorescence of the chlorophylls and the neutral lipid bodies that are stained by BODIPY have a yellow fluorescence signal. Under nitrogen-deplete conditions, there is significantly more BODIPY fluorescence indicating accumulation of neutral lipids that mainly consist of TAG molecules. Not all cells show the same neutral lipid content under nitrogen-deplete conditions. This heterogeneity between cells has been observed previously in *N*. *oleoabundans* [32,44]. This could be explained by the fact that, under constant light conditions, not every cell is in the same stage of the cell cycle when the TAG accumulation phase is induced. Individual differences in metabolic activity and internal nitrogen pool might determine whether cells start to produce TAG or not. The total fatty acid (TFA) content increased as a consequence of the increase in TAG when the cells are exposed to nitrogen-deplete conditions (Fig 1E). In the first 24 hours of nitrogen depletion, *N*. *oleoabundans* accumulates 12.1%±1.4 and 10.3%±0.8 of their dry weight as TAG molecules in fresh water and salt water conditions, respectively. The TFA and TAG content is therefore comparable for the salt water adapted cells compared to the fresh water culture, and the TAG fraction of the TFA content is the same under both conditions. In Fig 1F (or S1 Table) the fatty acid profiles are shown for both TFA and TAG relative to the total cell dry weight. No significant changes in fatty acid composition are observed when comparing fresh water with salt water conditions, indicating that the salt resistance of *N*. *oleoabundans* is unlikely to be a consequence of modifications of the plasma membrane lipid composition. This is different for *Dunaliella salina*, which induces fatty acid elongation and expression of their related genes under saline stress [45].

### 3.2 De-novo transcriptome assembly and annotation

For the *de novo* assembly, RNAseq samples were pooled, amounting to 496,158,724 reads yielding 30489 contigs with a N50 of 2372 bp. Using a lower bound in contig size of 500 bp, 18097 contigs remained with a total length of 30,540,822 bp. QUAST [36] predicted these contigs to encode 32,136 protein coding sequences (CDS) with a total length of 20,517,587 bp. InterProScan5 [46] subsequently identified the presence of conserved protein domains in 54% (15,306) of these CDS. Additionally, 7021 putative proteins could be associated with Gene Ontology (GO) terms, of which 83% (5861) were unique. Finally, 3249 putative proteins were associated with EC numbers, 834 of which were unique. All sequences of genes, proteins, and the mentioned annotation results can be found in S1 File. At this time, there are two published studies of N. oleoabundans grown under nitrogen-deplete conditions, one with transcriptomics data and the other with proteomics [47,48]. The experimental conditions are fundamentally different from our set-up, which makes it difficult to compare the results directly.

### 3.3 Compatible solutes

Compatible solutes are highly soluble low molecular weight molecules that can be accumulated to high concentrations without being toxic to the cell. They can protect cells against drought or saline stress by regenerating cellular osmotic homeostasis, relieving oxidative stress caused by Reactive Oxygen Species (ROS), and protecting membrane integrity and stabilization of enzymes or proteins [49]. Some examples of compatible solutes are, free amino acids, sugars, polyols and quaternary ammonium compounds (QAC). We used the transcriptomic landscape under stress (nitrogen-deplete) conditions in comparison to the unstressed conditions as proxy to identify compounds that may act as a compatible solute or otherwise might be involved in protecting the algae against salt stress. For some of these compounds identified with RNAseq data we were able to confirm their role by measuring the intracellular concentrations.

#### 3.3.1 Sugars

**Fig 2:**
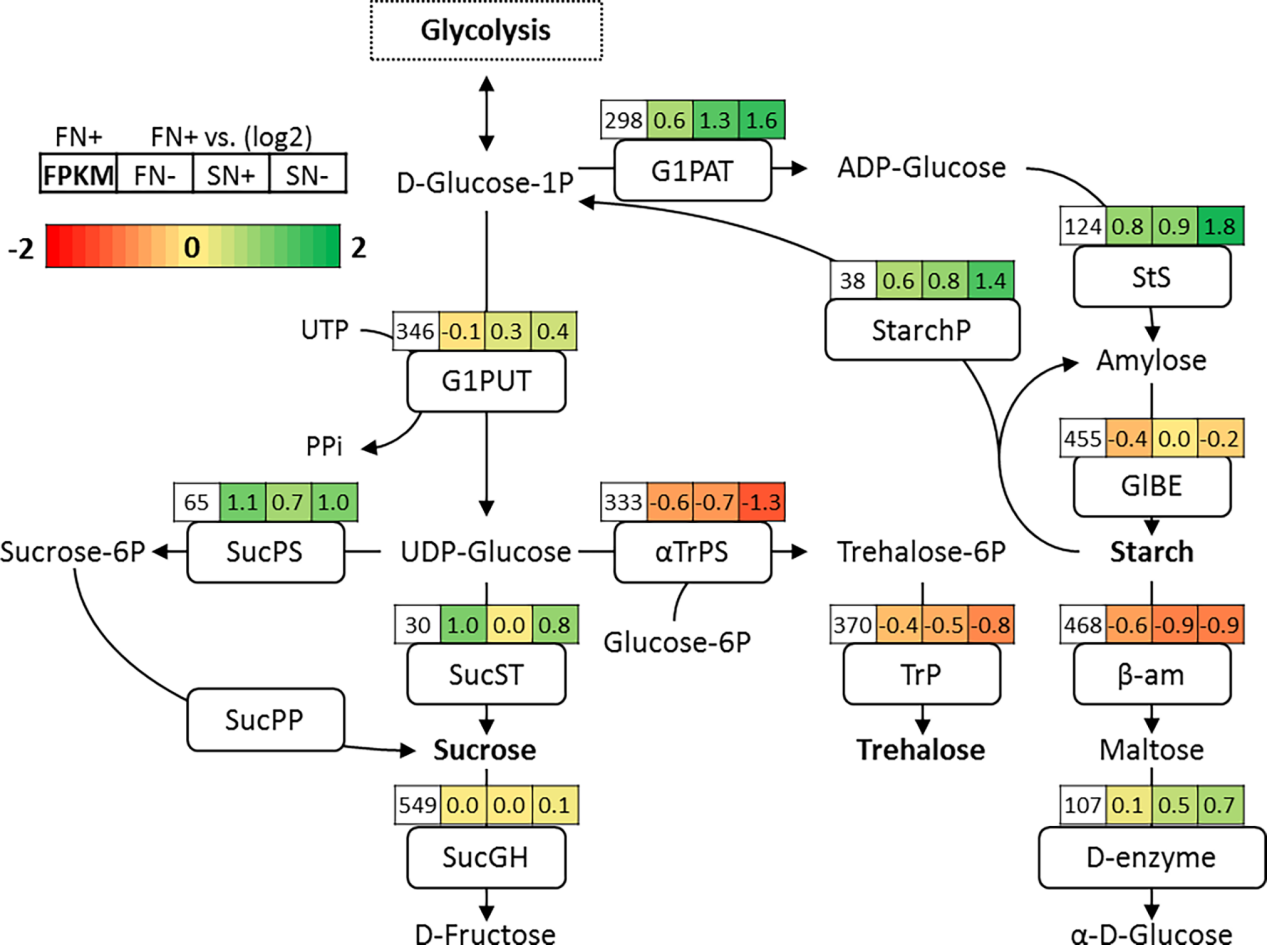
Biosynthesis pathway of sucrose and starch. The values that are shown in the following figures refer to the Fragments Per Kilobase of transcript per Million mapped reads (FPKM) for each condition. The white left-most box represents the FPKM value of the respective gene in the fresh water nitrogen-replete reference condition. This is followed by three colored boxes that represent the log2 fold change (LFC) of the other conditions compared to the reference condition. The order of the remaining three boxes are from left to right, FN-, SN+, SN- respectively. Abbreviations: G1PUT: glucose-1-phosphate uridylyltransferase; UTP: Uridine triphosphate. SucPS: sucrose-phosphate synthase. SucST: sucrose synthase. G1PAT: glucose-1-phosphate adenylyltransferase. αTrPS: alpha,alpha-trehalose-phosphate synthase. αTrS: alpha,alpha-trehalose synthase. TrP: trehalose-phosphatase. StS:starch synthase. GlBE: glycogen branching enzyme. SucPP: sucrose-phosphate phosphatase. SucGH: sucrose glucohydrolase. β-am: β-amylase. D-enzyme: 4-alpha-glucanotransferase. StarchP: Starch phosphorylase.

Several sugars are known to have a protective role in organisms experiencing different stress conditions [7]. Examples of osmoprotectant sugars are trehalose and sucrose. In most marine and freshwater cyanobacteria sucrose is synthesized from fructose-6-phosphate and UDP-glucose by the enzymes sucrose phosphate synthase (SucPS, EC:2.4.1.14) and sucrose-phosphate phosphatase (SucPP, EC:3.1.3.24) [50]. *Anabaena sp*. synthesizes sucrose in one step converting fructose and ADP or UDP glucose using sucrose synthase (SucST, EC:2.4.1.13) [51]. In *N*. *oleoabundans*, transcription of several genes in the sucrose biosynthesis pathway are up-regulated under saline growth conditions. Transcripts are considered differentially expressed when the FPKM values are at least log2 fold change (LFC) 0.59 (FC 1.5) compared to the reference condition. In this pathway, D-glucose-1P derived from glycolysis is converted into UDP glucose by the enzyme G1PUT which is marginally overexpressed under saline growth conditions. The conversion from UDP-glucose to sucrose is catalyzed by the enzyme sucrose synthase (SucST) and is strongly overexpressed under-nitrogen-deplete conditions (Fig 2).

The first step in the pathway that goes from UDP-glucose to sucrose via sucrose-6P catalyzed by SucPS is up-regulated under both salt water and nitrogen stressed cells. The second step catalyzed by sucrose–phosphate phosphatase (SucPP) is absent from the annotation. Sucrose can be degraded by sucrose glycohydrolase (SucGH) into D-fructose. Band et al. found that the main soluble carbohydrate that is accumulated in *N oleoabundans* experiencing a salt osmotic up-shock is sucrose [22]. Based on the sucrose levels measured and the expression levels of the genes involved, sucrose might be functioning as quick response to saline stress [22], but does not seem to be responsible for salt resistance in long term salt adapted cells. The NMR spectroscopy analysis revealed that sucrose concentrations may be increased under saline and nitrogen stress conditions (S2 Fig). Sucrose accumulation seems to be more of an overflow mechanism that results from nitrogen depletion.

Another sugar that is often found to be involved in salt resistance is trehalose. In high-temperature stressed yeast cells, trehalose concentrations are increased to protect enzymes from the elevated temperatures [52]. Trehalose is found in different microalgal species under saline stress conditions [53,54], but does not seem to be an osmoprotectant in *N*. *oleoabundans*, since key enzymes in this pathway are not transcriptionally up-regulated under saline conditions (Fig 2). This observation does however not completely exclude trehalose as a candidate for osmoprotection, because trehalose concentration was not analyzed directly and it could be that the flux toward this compound is not controlled at transcript level but on a metabolic level.

#### 3.3.2 Proline

Proline was already found to have many positive effects to cope for osmotic stress in bacteria, plants and algae [7,55–59]. We summarize the effects and pathway regulations for proline as found in diverse studies in S1 Text.

**Fig 3:**
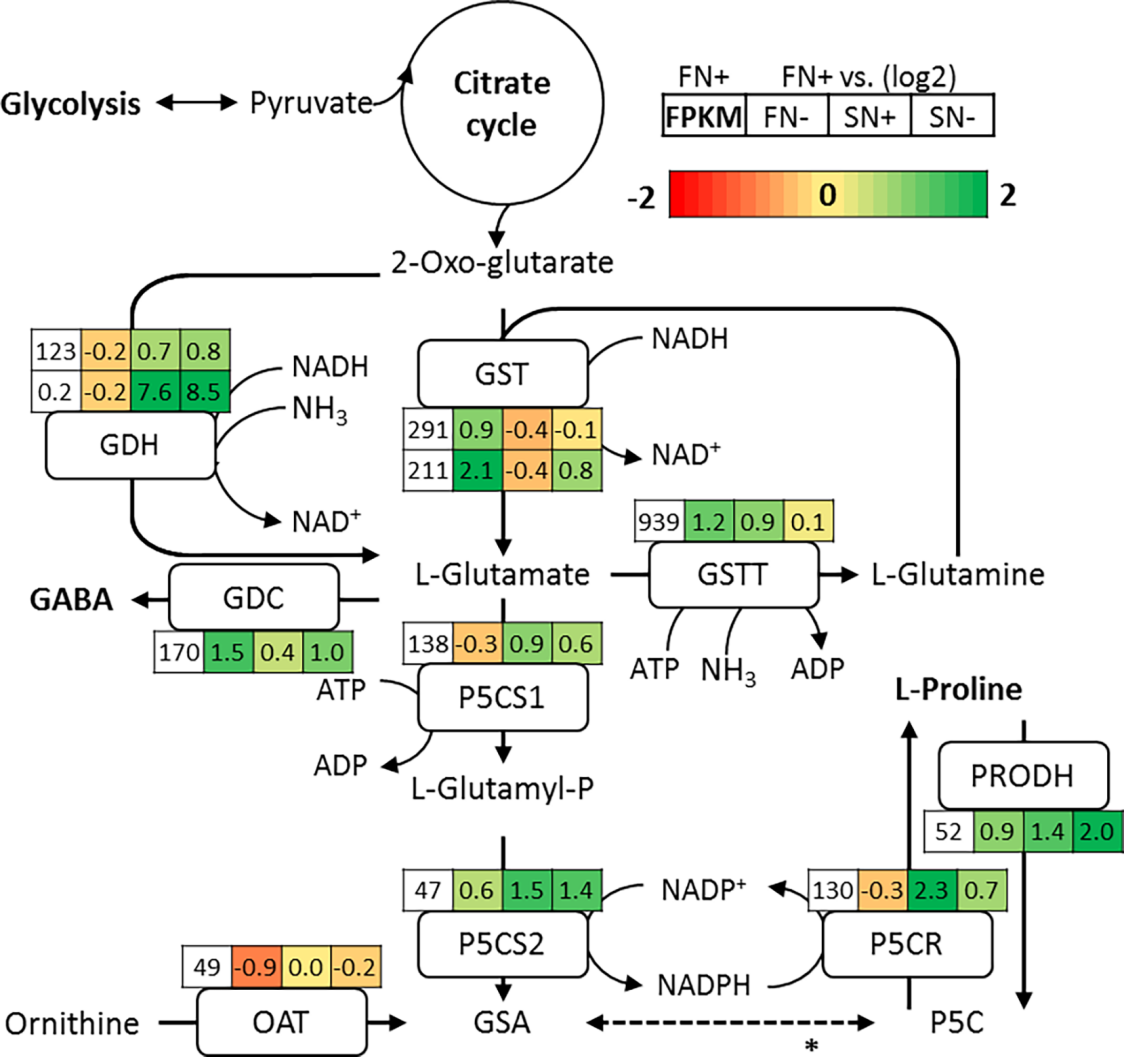
Proline and GABA biosynthesis pathway. For the figure legend refers to Fig 2. Abbreviations: GSA: glutamate-semialdehyde. P5C: L-1-Pyrroline-5-carboxylate. GABA: 4-aminobutanoate. P5CR: P5C reductase. P5CS1: glutamate-5-kinase. P5CS2: GSA dehydrogenase. GDH: glutamate dehydrogenase (EC:1.4.1.3/4). GST: glutamate synthase (EC:1.4.1.13/14). GSTT: glutamine synthetase. GDC: glutamate decarboxylase. OAT: ornithine aminotransferase. PRODH: Proline dehydrogenase. *This reaction occurs non-enzymatically.

In *N*. *oleoabundans*, many genes upstream of proline starting from the tricarboxylic acid (TCA) cycle are up-regulated under saline growth conditions. Based on the transcript levels it appears that the major route from the TCA cycle intermediate 2-oxo-glutarate towards L-glutamate is catalyzed via the enzyme glutamate dehydrogenase (GDH). There are two enzymes known that can catalyze this reaction: EC:1.4.1.3 and EC:1.4.1.4 using different co-factors. Both enzymes are up-regulated under saline growth conditions, the first by LFC 0.7 in SN+ and by LFC 0.8 in SN- and the second by much stronger LFC 7.6 (FC ~200) in SN+ and LFC 8.5 (FC ~350) in SN-(Fig 3). The enzyme complex P5CS1 and P5CS2 converts L-glutamate into L-glutamyl-P and GSA respectively. Both enzymes are strongly up-regulated under saline growth conditions. For the P5CS1 gene this is LFC 0.9 times and LFC 0.6 times for nitrogen-replete and -deplete saline growth conditions respectively. For the P5CS2 enzyme, the up-regulation under these conditions are LFC 1.5 and LFC 1.4 respectively. The alternative way to produce GSA by OAT from ornithine seems to be less important and only active under nitrogen-replete conditions and with slightly higher expression under salt water conditions (Fig 3). In contrast, the diatom *Fragilariopsis cylindrus* is primarily using the ornithine pathway to generate proline as a response to salt stress [60]. The final conversion from P5C to proline through the P5CR reaction, is markedly up-regulated under saline conditions. However, this overexpression is stronger in SN+ than SN- (LFC 2.3 and LFC 0.7, respectively) Furthermore, catabolism of proline is done by proline dehydrogenase (PRODH, EC:1.5.99.8) and is upregulated in all conditions in comparison to FN+. Most importantly, PRODH overexpression was stronger in SN- than SN+ (LFC 2.0 and LFC 1.4 respectively). Thus, based on this proline synthesis would be more upregulated and breakdown less upregulated under the SN+ as compared to the SN- condition.

Proline content was then studied for all four conditions by NMR spectroscopy, results are shown in S2 Fig. Although the differences between the duplicate measurements are large, still an increase in proline content can be observed for the SN+ condition, which is in agreement with the transcriptome results. The cause for the large difference between the duplicate measurements is likely due to the difficulty to disrupt the cell wall of *N*. *oleoabundans*. Furthermore, no increase in proline content is measured for the SN- condition. This could be due to the upregulated breakdown pathways under the SN- condition and the fact that in the SN- condition nitrogen, which is needed for proline synthesis, is absent.

To conclude, since proline levels are increased in salt water adapted cells and all genes involved in the proline biosynthesis are up-regulated, proline is most likely to be the primary mechanism in the saline resistance of *N*. *oleoabundans*. Furthermore, under nitrogen-deplete conditions, PRODH is up-regulated possibly to enable the recycling of nitrogen to use the nitrogen molecules from proline in other nitrogen requiring processes in the cell.

More detailed descriptions and analysis of Proline and other potential osmoregulatory mechanisms can be found in S1 Text.

### 3.4 Oxidative stress

**Fig 4:**
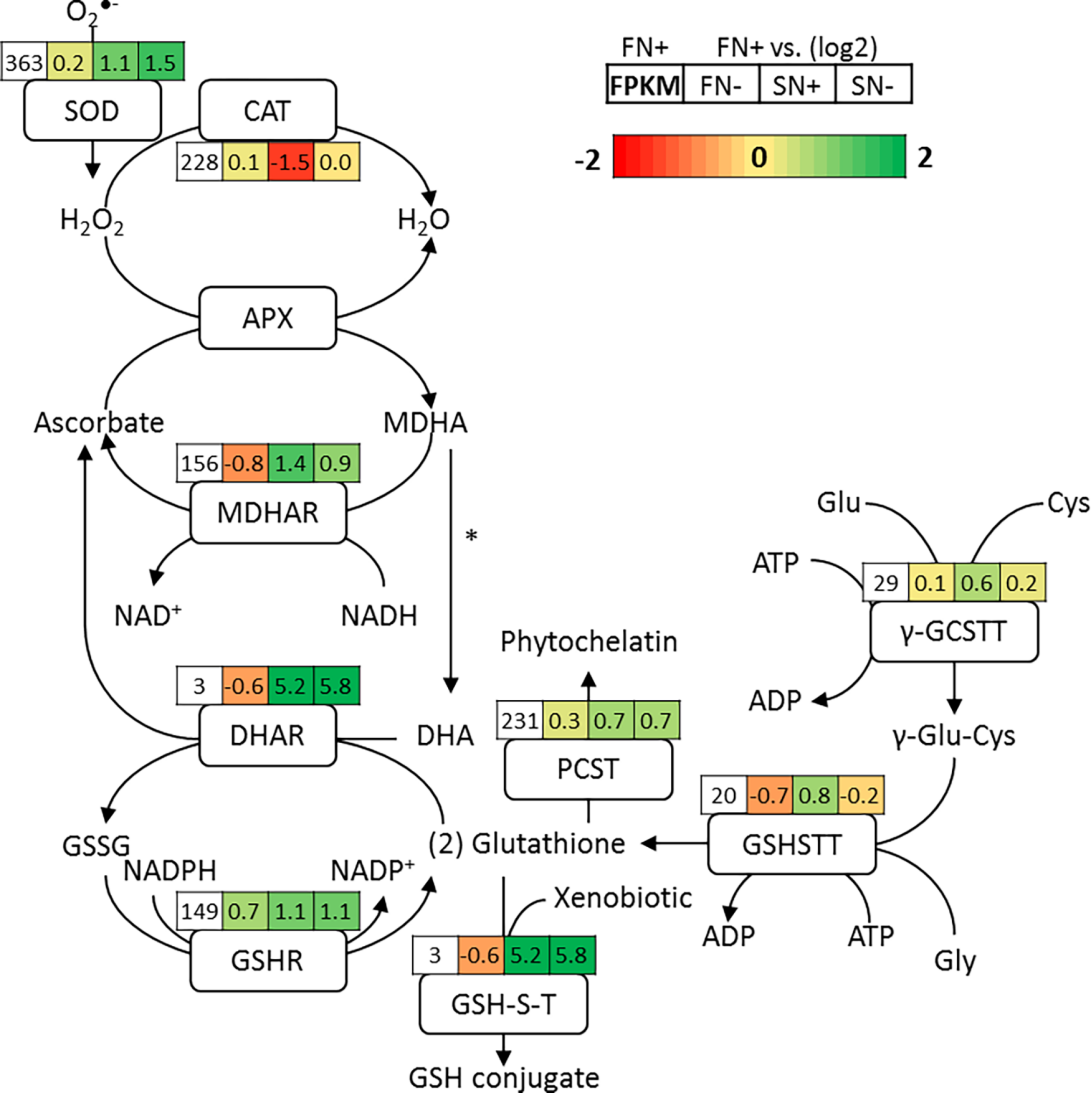
Glutathione biosynthesis pathway and glutathione-ascorbate cycle. For the figure legend refers to Fig 2. Abbreviations: SOD Superoxide dismutase; CAT Catalase; APX Ascorbate peroxidase (not annotated); MDHA monodehydroascorbate; MDHAR monodehydroascorbate reductase; DHA dehydroascorbate; DHAR dehydroascorbate reductase; GSH (reduced) Glutathione; GSSG oxidised glutathione; GSHR Glutathione reductase; Glu Glutamate; Cys Cysteine; Gly Glyceine; γ-GCSTT γ-glutamylcystein Synthetase; γ-Glu-Cys γ-glutamylcystein; GSHSTT Glutathione Synthetase; GSH-S-T glutathione S-transferase; PCST Phytochelatin synthase. * This reaction can occur enzymatically or non-enzymatically.

Under stress conditions ROS can be formed which need to be removed to prevent damage to the photosystems and other cellular equipment [61]. Glutathione (GSH) is a pivotal compound for many plant species and microalgae to scavenge ROS, such as superoxide hydrogen peroxide and lipid hydroperoxides, which can be accumulated under environmental and oxidative stress [62]. In addition to direct scavenging of ROS, GSH can also function as the reductant in the glutathione-ascorbate cycle which can alleviate ROS build up and provide protein protection. The tripeptide glutathione (GSH) is synthesized from glutamate and cysteine into γ-glutamylcysteine by γ-glutamylcysteine synthetase (γ-GCSTT EC:6.3.2.2) at the expense of one ATP. In the next step, another ATP molecule is needed to convert γ-glutamylcysteine and glycine into glutathione, by glutathione synthetase (GSHSTT EC:6.3.2.3) (Fig 4).

The oxidative stress can be relieved by antioxidant enzymes. First, superoxide can be converted into hydrogen peroxide by the superoxide dismutase (SOD; EC:1.15.1.1). SOD is highly overexpressed under saline conditions LFC 1.1 and LFC 1.5 in nitrogen-replete and depleted conditions respectively (Fig 4). Second, the toxic hydrogen peroxide can be converted to H_2_O by catalase (CAT, EC 1.11.1.6) or by ascorbate peroxidase (APX EC:1.11.1.11). CAT expression is stable under nitrogen-deplete conditions and strongly down regulated under nitrogen-replete saline conditions. Such a regulation of CAT suggests that APX is the preferred way to relieve the harmful radicals.

Unfortunately, APX could not be annotated in *N*. *oleoabundans* from the mRNA sequences. MDHA, which is produced by APX, needs to be converted back to ascorbate via two different ways. Enzymatically, by monodehydroascorbate reductase (MDHAR EC:1.6.5.4), or non-enzymatically by spontaneous disproportionation of two MDHA molecules resulting in ascorbate and dehydroascorbate (DHA) [63]. DHA will subsequently be converted back to ascorbate by dehydroascorbate reductase (DHAR EC:1.8.5.1) coupling two GSH molecules into glutathione disulfide (GSSG). Glutathione reductase (GSHR EC:1.8.1.7) can catalyze the reduction of GSSG back to two GSH molecules. The MDHA reductase gene is LFC 1.4 increased under saline replete conditions and LFC 0.9 increased under nitrogen-deplete conditions. DHA reductase was strongly up-regulated under salt water conditions to regenerate the ascorbate from DHA. DHAR was LFC 5.2 (FC 37) and LFC 5.8 (FC 56) up-regulated under nitrogen-replete and -deplete salt water growth conditions respectively. To recycle the GSH molecules, GSHR is up-regulated under salt water nitrogen-replete and -deplete conditions as well, namely LFC 1.1 and LFC 1.1 times respectively.

The efficiency of GSH is dependent on its concentration in the cell, which in turn depends on the activity of the GSH reductase enzyme, which determines the ratio between GSH and its oxidized form GSSG. As shown, all genes involved in the ascorbate-GSH cycle were up-regulated under salt water growth conditions, including the biosynthesis of GSH (Fig 4).

Another function of GSH is the detoxification of xenobiotics, compounds that have no significant nutritional function in cell metabolism, but do affect cellular homeostasis in too high concentrations, this in contrast to compatible osmolytes that do not interfere with metabolism when present in high concentrations. The xenobiotic molecules can be conjugated to GSH, by GSH-S-transferase (GSHT, EC:2.5.1.18), and transported to vacuoles to be detoxified. Under saline conditions this enzyme is strongly up-regulated, LFC 5.2 and LFC 5.8 under nitrogen-replete and -deplete saline conditions respectively. This indicates the likeliness of GSH conjugate formation and exclusion to relieve xenobiotic pressure under saline conditions (Fig 4). It is known in plants that GSHT is induced under salt and drought stress to reduce the ROS in plants and GSHT could also be involved in reducing the harmful byproducts of oxidative stress such as lipid peroxidation [64,65].

A third protecting feature of GSH is the formation of phytochelatins (PC) by the enzyme phytochelatin synthase (PCST EC:2.3.2.15). PCs are oligomers of GSH and are able to detoxify heavy metals by chelation of toxic ions [66,67]. The PC-ion complexes can be compartmentalized in the vacuole of plants where they can do no further harm. PC has also been found to be involved in stress responses other than to heavy metals. In the cyanobacterium *Anabaena doliolum* PCs are produced in response to UV-B radiation [68]. The cloning of the PC synthase gene of *Anabaena sp*. into *E*. *coli* increased the resistance of this bacterium to heat, metals, UV-B, salt, and herbicides [69]. Some plants have been shown to produce PCs in response to heat or salt stress [70,71]. The *N*. *oleoabundans* PCST gene is up-regulated in salt water medium under both nitrogen conditions (LFC 0.7), indicating that PCs are likely to be formed to protect *N*. *oleoabundans* against osmotic stress.

Based on the general increase in expression of the genes involved in the ascorbate glutathione cycle, it is very likely that *N*. *oleoabundans* is using this cycle to alleviate the pressure of ROS that arise under saline growth conditions and that the GSH derived conjugates and phytochelatins GSH oligomers are likely to be involved as well. This mechanism is not used to alleviate the ROS pressure under nitrogen stress, at least not under the tested conditions.

### 3.4 Starch and triacylglycerol accumulation

#### 3.4.1 Starch pathway

Starch is known to be transiently accumulated under nitrogen-deplete conditions in the beginning of the stress phase [30,44,72]. In this study, the sample time was 24 hours after nitrogen stress induction and it is expected that the genes involved in starch biosynthesis would be up-regulated in this early phase of nitrogen stress. The starch levels under the four different conditions are shown to be strongly increased in the first 24 hours after nitrogen depletion. Under fresh water conditions, the starch content increased from 10.5%±0.1 to 33.5%±0.4 when switched to nitrogen depletion and from 12.9%±01.1 to 39.5%±0.0 under nitrogen depletion in salt water conditions (Fig 1E). Interestingly, salt water adapted cells had a 1.2 times higher starch content compared to the fresh water adapted cells under both nitrogen-replete and -deplete conditions.

The genes encoding glucose-1-phosphate adenylyltransferase (G1PAT, EC:2.7.7.27) and starch synthase (StS, EC:2.4.1.21) are strongly up-regulated under nitrogen-deplete conditions and seems to be up-regulated by salt water as well (Fig 2). The G1PAT gene is LFC 0.6 times up-regulated in FN-, while this gene is up-regulated by LFC 1.3 in SN+ and by LFC 1.6 times SN-. Starch accumulation upon nitrogen depletion seems to be facilitated by transcriptional up-regulation of starch biosynthesis genes with the exception of the final step catalyzed by glycogen branching enzyme (GlBE, EC:2.4.1.18). Starch catabolism can be facilitated by different enzymes. β-amylase (β-am, EC:3.2.1.2) degrades starch by removing maltose units from the non-reducing ends of the chains. β-am was shown to be down-regulated under salt water and nitrogen-deplete conditions (Fig 2). Those results correlate with another nitrogen deplete transcriptomics study of N. oleoabundans [47] in which starch is also accumulated, the glucose branching enzyme (GlBE) is slightly down-regulated, and β-amylase is down-regulated. However, that study observed down-regulation of starch building enzymes G1PAT and StS, while in this study, we observed up-regulation of those genes. Unexpectedly, starch phosphorylase (StarchP, EC:2.4.1.1) and α-amylase (α-am, EC:3.2.1.1), two other starch degrading enzymes, are up-regulated under nitrogen-deplete conditions when there is accumulation of starch (S1 Table). While the change of expression is rather strong for StarchP, the change of expression of α-am is very small and cannot be considered significant. A possible explanation could be that during nitrogen depletion, starch turnover is accelerated with a faster anabolism than catabolism, while the starch degradation products are being redirected towards other sugars and TAG. Salt stress does seem to increase the starch content, which is possibly a result of reduced growth creating a surplus of energy that needs to be channeled from the photosystems [73].

#### 3.4.2 Triacylglycerol pathway

**Fig 5:**
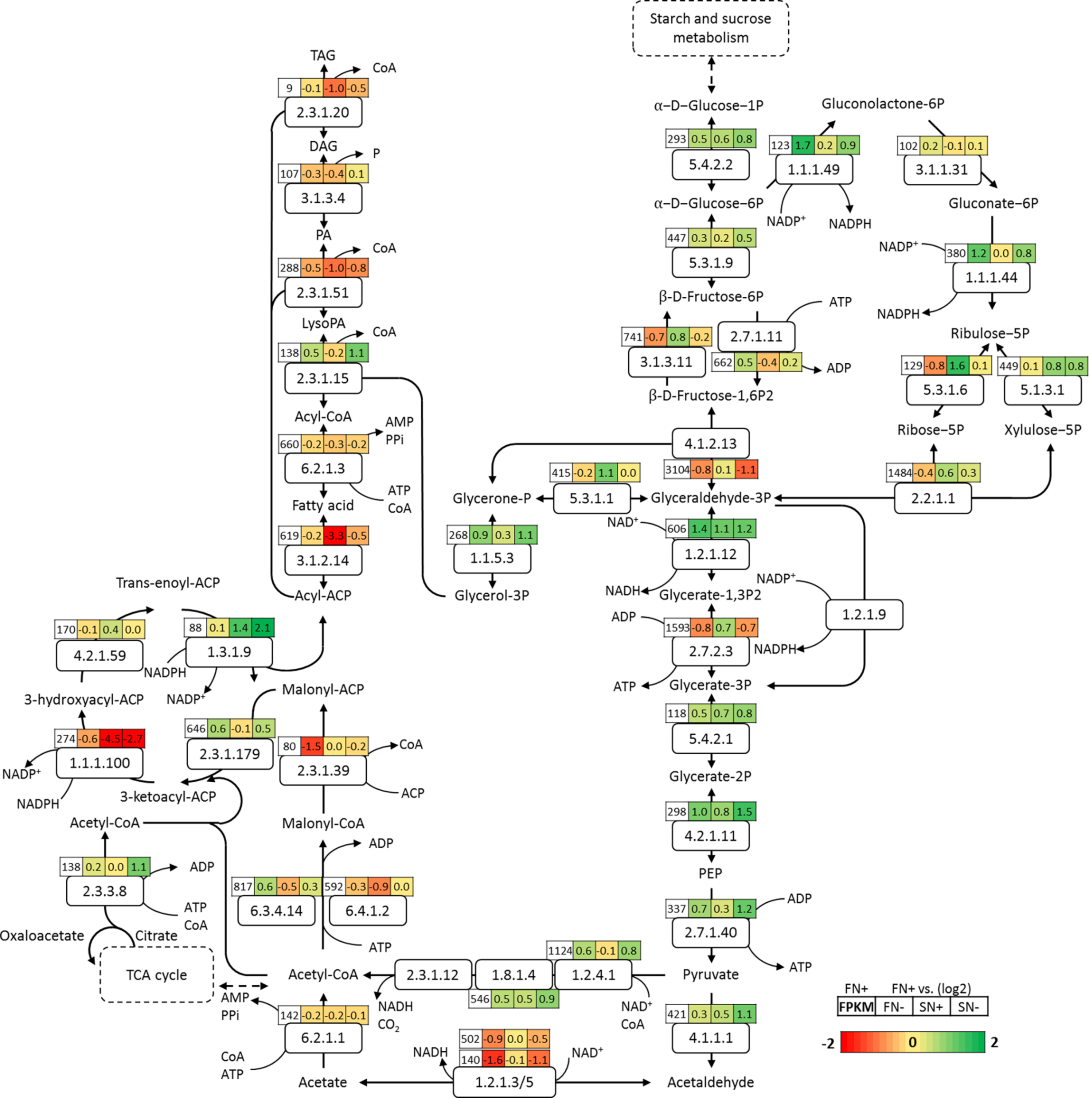
Carbon metabolism in *N*. *oleoabundans*. For the figure legend refers to Fig 2. Codes in the white boxes represent the corresponding EC numbers. Abbreviations: PEP phosphoenol pyruvate; LysoPA Lysophosphatidic acid; PA Phosphatidic acid; DAG 1,2-Diacylglycerol; TAG Triacylglycerol. EC:2.3.1.12 could not be annotated from the transcriptome of *N*. *oleoabundans*.

When oleaginous microalgae are exposed to unfavorable growth conditions such as nitrogen-deplete conditions, cells start to accumulate triacylglycerol (TAG). These neutral glycerolipids are composed of a glycerol backbone with three fatty acid molecules attached. The fatty acid biosynthesis takes place in the chloroplasts and starts downstream of the glycolysis with the conversion of acetyl-CoA to malonyl-CoA by the heteromeric enzyme complex acetyl-CoA carboxylase (ACCase). The acetyl-CoA pool can be supplied in two different ways. The first way is via pyruvate and the pyruvate dehydrogenase complex, in which the first two steps are up-regulated under nitrogen-deplete conditions in *N*. *oleoabundans* (EC:1.2.4.1 and EC:1.8.1.4, see Fig 5). Another way is via the TCA cycle in which citrate can be converted into acetyl-CoA and oxaloacetate by ATP citrate lyase (EC:2.3.3.8) [74]. The *N*. *oleoabundans* ATP citrate lyase is up-regulated under nitrogen-deplete conditions and it is likely that this pathway is involved in supplying part of the acetyl-CoA.

ACCase catalyzes an important step in fatty acid biosynthesis and overexpression of this enzyme resulted in increased lipid contents in *Arabidopsis* [75], but not necessarily in microalgae [76]. Over expression of the ACCase complex is challenging because of the multigene-encoded enzyme complex and post-translational modifications [77,78]. Next, an acyl-carrier protein (ACP) is exchanged for the CoA moiety by malonyl-CoA:ACP transacylase giving rise to a malonyl-CoA molecule. Malonyl-CoA enters the heteromultimeric fatty acid synthesis (FAS) cycle where it is extended with two carbon atoms per repetitive cycle to usually 16 or 18 carbon long acyl-ACP groups.

In this study, two subunits of the enzyme complex ACCase could be identified, biotin carboxylase (EC: 6.3.4.14) and biotin carboxyl carrier (EC:6.4.1.2). The biotin carboxylase subunit is up-regulated under nitrogen-deplete conditions, and the biotin carboxyl carrier has little (fresh water) to no (salt water) down-regulation under nitrogen-deplete conditions (Fig 5). These results thus show that the biotin carboxylase is the main regulator for ACCase complex which is in agreement with the results from both aforementioned N. oleabundans studies [47,48].

Our results show that the third reaction of the FAS cycle did not display any changes in expression (Fig 5), while the first (EC:2.3.1.179) and last (EC:1.3.1.9) step of the FAS cycle displayed up-regulation: the first step seems to be up-regulated due to nitrogen depletion, whereas the last step is strongly up-regulation due to salt water conditions. In contrast, the second reaction of the FAS cycle displayed light down-regulation due to nitrogen depletion and displayed very strong down-regulation due to salt water. In agreement with the other transcriptome study of *N*. *oleoabundans* [47], Acyl-ACP desaturase (AAD) was found to be significantly up-regulated, which also correlates with the very similar change in fatty acid profile in that study being mainly an increase in C18:1. Since the TAG content is strongly increased in the first 24 hours of nitrogen starvation (Fig 1), it was expected that transcription of the genes encoding the enzymes involved in the TAG synthesis pathway would be up-regulated under nitrogen-deplete conditions. This was not the case with the exception of the glycerol 3-phosphate acyltransferase (EC:2.3.1.15) gene, which showed a LFC 0.5 and LFC 1.1-under fresh water and salt water nitrogen-deplete conditions respectively (Fig 5). This enzyme links glycolysis and the TAG synthesis pathway by attaching the first acyl-ACP molecule to sn-Glycerol 3-phosphate resulting in lysophosphatidic acid. Glycerol-3-phosphate can be formed from glycerol via glycerol kinase (EC:2.7.1.30) or from dihydroxyacetone phosphate via glycerol-3-phosphate dehydrogenase (EC:1.1.5.3). The latter seems to be the case in *N*. *oleoabundans* since transcription of the gene for this enzyme is strongly up-regulated under nitrogen-deplete conditions. The overexpression of a yeast glycerol-3-phosphate dehydrogenase in rape seed, resulted in a 40% increase of the seed oil content, indicating that the available glycerol backbones are a limitation in TAG accumulation in rape seed [79]. To ensure sufficient supply of G3P, transcription of triose-phosphate isomerase (EC:5.3.1.1), the enzyme that converts glycerone-P (Dihydroxyacetone phosphate, DHAP) to glyceraldehyde-3-P, is reduced under nitrogen-deplete conditions (Fig 5). Chen et al. described the increase of DHAP, G3P and glycerol in *Arabidopsis* when triose-phosphate isomerase was knocked out [80]. A similar regulatory mechanism was found in microalgae [81].

The last step from diacylglycerol (DAG) to triacylglycerol (TAG) is catalyzed by diacylglycerol O-acyltransferase (DGAT, EC:2.3.1.20). This enzyme is down regulated in *N*. *oleoabundans* under all tested conditions compared to the fresh water replete conditions. DGAT has been extensively studied since it is regarded as one of the most important rate limiting steps in TAG biosynthesis. Over expression of this gene or several subunits have resulted in very different outputs. In *Phaeodactylum tricornutum* there have been very promising results, resulting in higher levels of TAG synthesis, but in the green alga *C*. *reinhardtii* there have not been consistent increases in TAG content in combination with DGAT over expression [82–85]. The fact that DGAT does not seem to be over expressed under nitrogen-deplete conditions is surprising, since TAG accumulation is occurring in *N*. *oleoabundans* under these circumstances. In this study only one DGAT gene could be identified and it is known from other species that there are several genes that encode for DGAT enzymes and that they have very different expression profiles as a response to nitrogen deprivation [73,86,87]. More research needs to be done to understand and identify the different DGAT enzymes in *N*. *oleoabundans*.

As can be seen in Fig 5, most of the genes involved in energy and carbon metabolism are up-regulated under nitrogen-deplete conditions. Under salt conditions, transcription of genes involved in glycolysis, TCA cycle, and pentose phosphate pathway (PPP) were increased. This can be explained by the need for sufficient carbon precursors such as acetyl-CoA and ATP. Furthermore, it was already hypothesized [72] that PPP might be used to redirect the starch degradation products towards TAG biosynthesis. In the PPP, the transcripts for the NADPH generating enzymes are strongly up-regulated to generate the required NADPH that is needed for TAG biosynthesis and to scavenge ROS.

To maintain high levels of TAG molecules, the rate of TAG catabolism should be low [88]. The gene triacylglycerol lipase (EC:3.1.1.3) is highly expressed under nitrogen-replete fresh water conditions (FPKM: 1734). The expression is down regulated under nitrogen-deplete and salt water conditions, LFC -0.3, LFC -0.9, and LFC -0.6 for fresh water nitrogen-depleted, salt water nitrogen-replete and depleted conditions respectively (S1 Table).

## 4 Conclusion

To assess the metabolic and transcriptomic response to nitrogen and salt stress, *N*. *oleoabundans* was cultured under four different conditions being fresh water and saline water both under nitrogen-replete and nitrogen-deplete conditions. The most likely compatible solute in *N*. *oleoabundans* is proline because under saline conditions, transcripts involved in proline biosynthesis are up-regulated and NMR indicated that proline is accumulated in the cells. Next to this transcriptome analysis shows that anti-oxidant pathways, like the ascorbate GSH cycle, GSH-conjugation, PC formation and GSH itself, are upregulated under saline conditions, probably protecting the cells from oxidative stress occurring under this condition. Unlike other microalgae, the plasma membrane lipid composition of *N*. *oleoabundans* was not adjusted in salt water adapted cells. Under Nitrogen-deplete conditions both starch and TAG were accumulated at both fresh water and salt water conditions. With the overall gene expression, we could explain how starch is accumulated. However, no strong correlation was found between accumulation and gene expression in the TAG pathway. Nevertheless, we identified two genes as promising targets to enhance TAG production: glycerol-3-phosphate acyltransferase and glycerol-3-phosphate dehydrogenase. Not only because they link the glycolysis to the TAG biosynthesis pathway, but also because of their expression profile. Overall, the results of this study can be used to develop strategies to enhance salt resistance of *N*. *oleoabundans* and other industrially relevant microalgal strains and ultimately help to develop a competitive feasible large-scale production of TAG for feed and bio-fuels, while reducing the dependence on precious fresh water resources and arable land.

## Supporting information

S1 Fig Set up of the cultivation experiment. Panel A: Representation of the four different environmental conditions that were examined in this study. Panel B: Cultivation set up. Two cultures were grown in fresh water in nitrogen replete conditions. The dotted line indicates the moment of medium replacement. One culture remained nitrogen replete, the other culture was exposed to nitrogen depleted medium. The same regime was applied to salt water adapted cultures. The whole experiment was conducted in duplicate. This method results in comparable growth conditions (similar lighting and time of nitrogen depleted conditions.
(TIF)

S2 Fig NMR spectroscopy showing metabolite concentrations under different conditions. Samples were measured in duplicate. The height of the bars represents the average of the two independent measurements. Error bars represent distance of the sample values to the average value.
(TIF)

S3 Fig NMR Quantitative variation in the ^1^H NMR spectra from all four conditions in duplicate. Spectra are derived from ethanol–water extracts of individual samples of FN+ fresh water replete, FN- Fresh water nitrogen depleted, SN+ Salt water replete, SN- salt water nitrogen depleted conditions. The numbers I and II indicate duplicates.
(TIF)

S1 Text Complementary information. (DOCX)

S1 Table Fatty acid composition of total fatty acid and triacylglycerol lipids expressed as fraction of dry cell weight. Values are the average of the biological duplicates.
(DOCX)

S2 Table FPKM values and fold change for all transcripts discussed in this manuscript. (DOCX)

S3 Table FPKM values and fold change for transcripts encoded for ATPases. (DOCX)

S1 File Excel sheets file containing sequence of genes and proteins, and the results of annotation for GO+EC, TargetP, PredAlgo, and WolfPSORT. (XLSX)

## Abbreviations

ACP: Acyl-carrier protein
CDS: Coding sequence
CoA: Co-enzyme A
DAG: Diacylglycerol
DGAT: Diacylglycerol O-acyltransferase
FAMEs: Fatty acid methyl ethers
FC: Fold Change
FPKM: Fragments per kilobase per million mapped reads
GABA: γ-Aminobutyrate
LFC: log2 Fold Change
NMR: Nuclear magnetic resonance
PC: Phytochelatins
PSI: Photosystem I
QAC: Quaternary ammonium compound
ROS: Reactive oxygen species
TAG: Triacylglycerol
TFA: Total fatty acid
