# Supplementary figures and images for: Neochloris oleoabundans is worth its salt: Transcriptomic analysis under salt and nitrogen stress
Source: PLoS One. 2018 Apr 13;13(4):e0194834. doi: 10.1371/journal.pone.0194834 (PMC5898717; doi:10.1371/journal.pone.0194834)

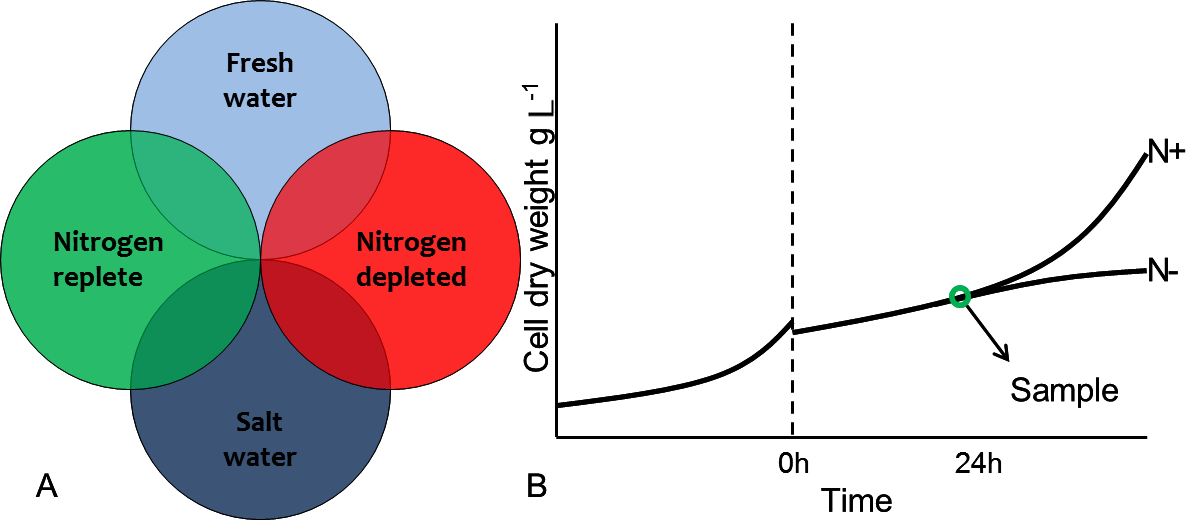

Supplement: S1 Fig — Panel A: Representation of the four different environmental conditions that were examined in this study. Panel B: Cultivation set up. Two cultures were grown in fresh water in nitrogen replete conditions. The dotted line indicates the moment of medium replacement. One culture remained nitrogen replete, the other culture was exposed to nitrogen depleted medium. The same regime was applied to salt water adapted cultures. The whole experiment was conducted in duplicate. This method results in comparable growth conditions (similar lighting and time of nitrogen depleted conditions. (TIF) [file pone.0194834.s001.tif]

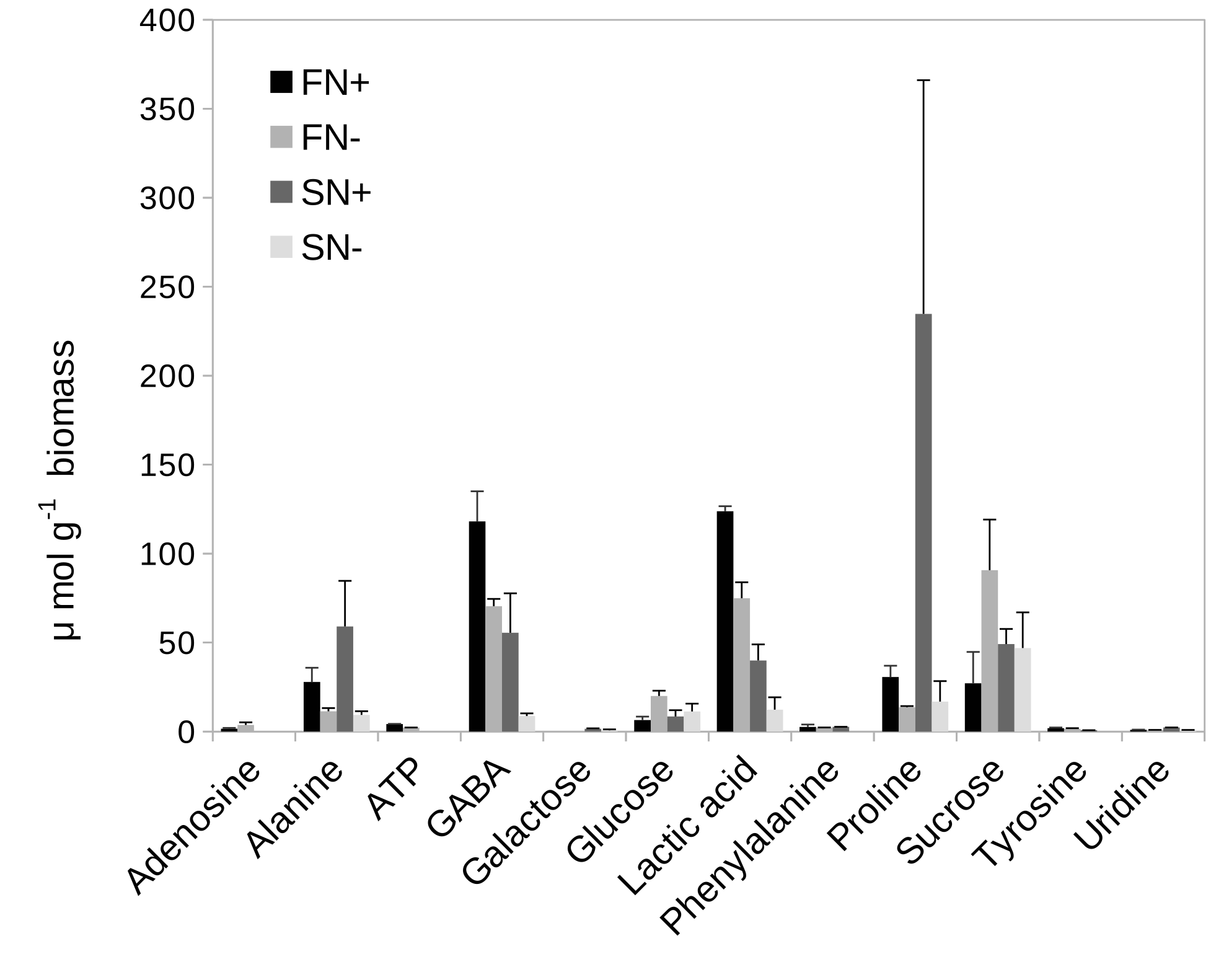

Supplement: S2 Fig — Samples were measured in duplicate. The height of the bars represents the average of the two independent measurements. Error bars represent distance of the sample values to the average value. (TIF) [file pone.0194834.s002.tif]

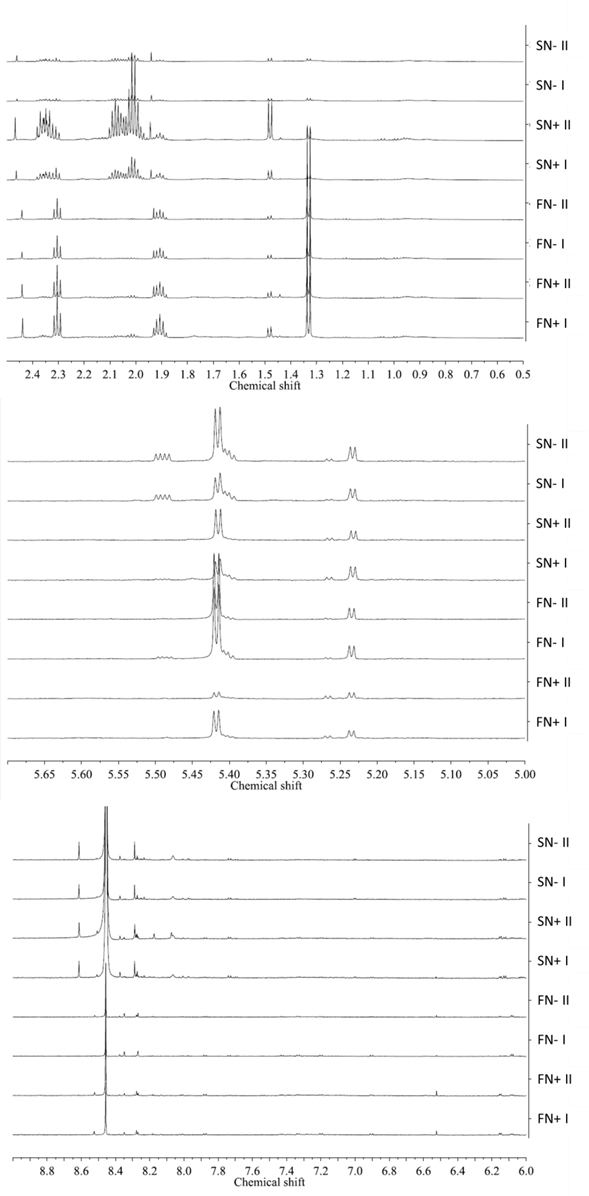

Supplement: S3 Fig — Spectra are derived from ethanol–water extracts of individual samples of FN+ fresh water replete, FN- Fresh water nitrogen depleted, SN+ Salt water replete, SN- salt water nitrogen depleted conditions. The numbers I and II indicate duplicates. (TIF) [file pone.0194834.s003.tif]
