# Supplementary material for: Neochloris oleoabundans is worth its salt: Transcriptomic analysis under salt and nitrogen stress
Source: PLoS One. 2018 Apr 13;13(4):e0194834. doi: 10.1371/journal.pone.0194834 (PMC5898717; doi:10.1371/journal.pone.0194834)
Supplement: S1 Text — (DOCX) [file pone.0194834.s004.docx]

# Proline

Proline is a proteinogenic imino acid that has strong conformational rigidity and is involved in primary metabolism. In bacteria proline levels are known to be related to the osmotic stress response [[1](#_ENREF_1)][. In plants proline can function as a chemical chaperone in cell metabolism preventing protein aggregation when cells are exposed to extreme conditions such as high temperatures, heavy metals or osmotic stress, so that enzymes can function normally](#_ENREF_1) [[2](#_ENREF_2),[3](#_ENREF_3)][. Proline is known to be able to take action as an active osmolyte for osmotic adjustment as well. Proline also has an antioxidant function by reducing the harmful effect of singlet oxygen and hydroxyl radicals on photosystem II (PSII)](#_ENREF_3) [[4-6](#_ENREF_4)][. Another important function of proline is maintaining the redox balance preventing photoinhibition or loss of photosynthetic efficiency by keeping the NADPH:NADP](#_ENREF_4)^+^ ratio low enabling electron flow between reaction centers in the photosystems [[7](#_ENREF_7)][. Proline can reduce the peroxidation of lipids in](#_ENREF_7) *Chlorella vulgaris* and *Chlamydomonas reinhardtii* cells exposed to heavy metals and increase the activity of pyrroline-5-carboxylate synthase (P5CS, EC:1.2.1.88) and, in parallel, increase the intracellular reactive nitrogen species NO level [[8-10](#_ENREF_8)][. When proline accumulation was enhanced in transgenic](#_ENREF_8) *C. reinhardtii* by over expressing a P5CS gene from moth bean, the levels of free radicals were reduced in reaction to the toxic metal cadmium [[11](#_ENREF_11)][.](#_ENREF_11)

In order to synthesize proline, in many organisms including plants and microalgae, inorganic nitrogen is first reduced to ammonium before it is used in the biosynthesis of several nitrogen containing metabolites such as amino acids [[12-14](#_ENREF_12)]. Ammonium is assimilated into glutamate or glutamine, which functions as the precursor and donor for the amino acids proline and arginine. In higher plants proline is synthesized in the cytosol mainly from reduction of glutamate to glutamate-semialdehyde (GSA) by the bifunctional enzyme P5CS [[15](#_ENREF_15),[16](#_ENREF_16)]. In some organisms, this enzymatic step is carried out by two separate enzymes, glutamate-5-kinase (P5CS1, EC:2.7.2.11) and glutamate-5-semialdehyde dehydrogenase (P5CS2, EC:1.2.1.41). Based on the transcriptome data this is also the case in *N. oleoabundans* (Fig3) and based on the genome of the model species *C. reinhardtii* [[17](#_ENREF_17)]. Unlike plants, microalgae and other microorganisms are not able to directly convert glutamate into GSA and that can be problematic, since the intermediate L-glutamyl 5-phosphate is rapidly cyclized into 5-oxoproline and phosphate. This issue is resolved by the formation of a complex of P5CS1 and P5CS2 to prevent the early cyclization of the labile intermediate L-glutamyl 5-phosphate in bacteria [[18](#_ENREF_18)], and a similar mechanism is believed to exist in microalgae [[17](#_ENREF_17)]. Subsequently GSA is spontaneously converted to pyrroline-5-carboxylate (P5C) and further reduced by the enzyme P5C reductase (P5CR, EC:1.5.1.2) to proline. These final two steps are found in all organisms studied. Another way to accumulate proline is by converting ornithine to GSA and P5C by the enzyme ornithine-delta-aminotransferase (OAT, EC:2.6.1.13) (Fig3).

# Lactic acid

As mentioned, CAT is downregulated in SN+ and stable in nitrogen deplete conditions. This indicates that it is not participating to remove oxidative stress due to salinity. CAT was found to improve acetic acid tolerance in yeast [[19](#_ENREF_19)], and our NMR results suggested that lactic acid was in lower concentration in salt water (S2 Fig). The lower concentration of lactic acid is to be expected as it is categorized as a ROS and therefor is known to induce oxidative stress caused by salt water. Our annotation did not successfully annotate the lactate dehydrogenase (1.1.1.27), but a manual annotation revealed one potential candidate gene (data not shown). This gene only displayed significant changes in the form of down-regulation in nitrogen deplete condition with no significant changes in salt water. This transcriptional regulation of fresh water nitrogen deplete does not fit our measurement which display a non-significant increase of lactic acid. The general incapacity to annotated the surrounding reactions did not allow us to form any speculations to explain this depletion of lactic acid on the transcription level. The most suitable explanation is that lactate accumulation in fresh water could be the production through overflow metabolism [[20](#_ENREF_20)]. Furthermore, due to salt water, the increased demand in ATP (partially due to GSH mechanisms) reinforces a general stress response, which results in a limited overflow metabolism and ultimately results in lessen quantities of lactic acid [[21](#_ENREF_21)].

# **Glycine** betaine

The use of glycine betaine (GB) as an osmoprotectant is not widespread throughout the plant kingdom. Rice, tobacco, Arabidopsis, and mustard do not produce GB naturally [[22](#_ENREF_22),[23](#_ENREF_23)][. Transgenic plants that are able to accumulate GB, have their salt resistance increased](#_ENREF_20) [[24](#_ENREF_24)][, indicating that GB accumulation can be an effective solution to counter osmotic stress resulting from saline environments. The freshwater cyanobacterium](#_ENREF_21) *Synechococcus sp.* is lacking the pathway to accumulate GB from choline. Transgenic strains that are complemented with the necessary genes from *E. coli* accumulated GB and had a higher resistance to salt water than the wild type when in combination with exogenous supply of choline, partly owing to stabilization of photosystem II [[25](#_ENREF_25)][.](#_ENREF_22)

GB is synthesized by converting choline into betaine aldehyde. In plants, this step occurs in the chloroplast and is catalyzed by the enzyme choline monooxygenase (EC:1.14.15.7) which was not found in *N. oleoabundans*. In many bacteria and mammals, the membrane bound enzyme choline dehydrogenase (CholDH, EC:1.1.99.1) or soluble enzyme choline oxidase (EC:1.1.3.17) is responsible for this step. The latter is not found *in N. oleoabundans*. CholDH is present in *N. oleoabundans* and its expression is strongly down-regulated under nitrogen-deplete conditions (S1 Table). This can be explained by the fact that the lack of nitrogen is resulting in down-regulation of the betaine pathway to use the nitrogen elsewhere. The second step from betaine aldehyde to betaine is catalyzed by betaine-aldehyde dehydrogenase (EC:1.2.1.8) and seems to be universal in plants bacteria and animals. This enzyme was not annotated for *N. oleoabundans*. An alternative pathway is known in bacteria using direct N-methylation of glycine via sarcosine. It is possible that betaine plays a role in osmoregulation in *N. oleoabundans*, but there is no strong evidence based on gene expression. In addition, a direct measurement of betaine, which was not possible with our NMR method, would also clarify this.

# Dimethylsulfoniopropionate (DMSP)

DMSP is a sulfonium compound that is known to be accumulated in several marine microalgae and some plants [[26](#_ENREF_26),[27](#_ENREF_27)][. DMSP functions as a compatible solute similar to betaine-like osmolytes, but has the advantage that the compound does not contain nitrogen and could therefore be used as an osmoprotectant under nitrogen limiting conditions. The biosynthesis of DMSP is different in microalgae and plants. Higher plants produce DMSP from S-methylmethionine while in the microalgae](#_ENREF_24) *Enteromorpha intestinalis* it was discovered that methionine is the precursor compound [[26](#_ENREF_26)][.](#_ENREF_23)

Methionine is transaminated to 4-methylhio-2-oxobutyrate (MTOB) and reduced to 4-methylhio-2-hydroxybutyrate (MTHB). MTHB is S-methylated into 4-dimethylsulfonio-2-oxobutyrate (DMSHB) which is oxidatively decarboxylated to DMSP [[28](#_ENREF_28)][. The first step from methionine to MTOB can be catalyzed by two aminotransferases (EC:2.6.1.5 EC:2.6.1.57). Both enzymes are annotated in](#_ENREF_25) *N. oleoabundans* and transcriptome data suggest that they are up-regulated in salt water growth conditions. The first aminotransferase is up-regulated LFC 0.5 and LFC 0.1 under salt water nitrogen-replete conditions and the second aminotransferase is up-regulated LFC 0.9 and LFC 0.6 under salt water nitrogen-replete conditions (S1 Table). Evidence for the existence of other downstream enzymes could not be extracted from the transcriptome data. There is still not much known about the exact enzymes involved in the DMSP biosynthesis pathway, making it hard to study transcript levels of genes involved in this pathway. The nitrogen rich amino acids will be reshuffled and recycled upon nitrogen depletion by transamination reactions. These transamination reactions can promote DMSP synthesis, since the first step in DMSP synthesis is the transamination of methionine to MTOB [[29](#_ENREF_29)][. It is difficult to draw conclusion on the role of DMSP as potential osmoprotectant in](#_ENREF_26) *N. oleoabundans*. Just like betaine, the transcriptome requires a better annotation and our NMR method could not measure DMSP.

# γ-Aminobutyrate

In *N. oleoabundans,* the γ-Aminobutyrate (GABA) content seems to decline in proportion to the amount of stress experienced (S2_Fig). The highest GABA concentration is found in nitrogen-replete fresh water conditions and the lowest content is found in salt water nitrogen-deplete conditions. This is not in correspondence with previous studies that investigated plant species, where GABA is found to be a protective osmolyte and can be accumulated in high amounts. Based on the NMR analysis, GABA does not seem to be of importance in the saline resistance of *N. oleoabundans*. The enzyme responsible for the conversion of L-Glutamate to GABA, glutamate decarboxylase (EC:4.1.1.15), is differentially expressed, but there is more expression of this gene under nitrogen-deplete conditions than under saline conditions (Fig3).

# ATPases

Transmembrane adenosinetriphosphatase (ATPases) are a class of enzymes (EC:3.6.1.3) that use the energy released from dephosphorylation of ATP into ADP and free phosphate to transport ions across a membrane against a concentration gradient, thus enabling reactions that would not occur spontaneously. These active transmembrane pumps are able to import nutrients into the cell or exclude toxic compounds from the cell. The enzyme group ATPases is a diverse generalized name for active transmembrane pumps. In this study 52 ATPases were found to be differentially expressed under the tested conditions. Around half of these transcripts seem to be correlated to salt water stress ranging from LFC 8.3 increase to LFC -9.2 decrease (S2 Table). An example of an ATPases known to be involved in osmotic regulation is the Na^+^-pump ATPase in *Dunaliella maritima* located in the plasma membrane [[30](#_ENREF_30)][. In](#_ENREF_27) *N. oleoabundans* the Na^+^/K^+^ pump (EC:3.6.3.9) is strongly up-regulated under salt conditions and even more in combination with nitrogen depletion (S2 Table). This pump is normally exclusively found in animal cells and not in plant or microalgal cells. It is likely that this transcript was wrongly annotated and is actually a different ATPase with close sequence similarity to the animal like Na^+^/K^+^ pump. The H^+^ exporting ATPase (EC:3.6.3.6) was found to be responsible for proton-pumping pyrophosphate into polyphosphate bodies in *C. reinhardtii* [[31](#_ENREF_31)][. This ATPase is up-regulated in](#_ENREF_28) *N. oleoabundans* under salt and nitrogen stress and enables the cell to decrease the acidity of the cytoplasm caused by stress, by pumping H^+^ ions and cations such as Ca^2+^ to vacuoles. Other ions that need to be actively excluded from the cell under salt stress are Mg^2+^ and Ca^2+^. These cations are actively transported from the cell to the surrounding environment by the Mg^2+^-translocating ATPase (EC:3.6.3.2) and Ca^2+^ ATPase (EC: 3.6.3.8), respectively. Both enzymes are not found to be up-regulated under any of the conditions tested in *N. oleoabundans*.

# Polyol pathway

A polyol is an alcohol containing several hydroxyl groups and polyols are found in many organisms. Examples of polyols are glycerol, mannitol, and sorbitol. The water-like hydroxyl groups in the polyols can prevent dehydration of molecules under salt water [[32](#_ENREF_32)][. Many plant and fungal species use this trick to sustain metabolic homeostasis and to overcome the negative effects of osmotic stress](#_ENREF_29) [[33](#_ENREF_33)][. Some microalgae accumulate polyols in response to osmotic stress. Different](#_ENREF_30) *Dunaliella* species are known to be resistant to saline conditions because of the accumulation of glycerol [[34](#_ENREF_34),[35](#_ENREF_35)][. Band et al. found that glycerol levels are not increased in salt shocked](#_ENREF_32) *N. oleoabundans* cultures [[36](#_ENREF_36)][. Also, in this study, there is no evidence for assigning the saline resistance of](#_ENREF_33) *N. oleoabundans* to glycerol or any other polyol. There is no evidence for polyol accumulation based on the gene expression of genes involved in the pathways for compounds such as sorbitol, mannitol, glycerol or any other polyol.

# References

1. Csonka LN, Gelvin SB, Goodner BW, Orser CS, Siemieniak D, et al. (1988) Nucleotide sequence of a mutation in the proB gene of Escherichia coli that confers proline overproduction and enhanced tolerance to osmotic stress. Gene 64: 199-205.

2. Sharma P, Shanker Dubey R (2005) Modulation of nitrate reductase activity in rice seedlings under aluminium toxicity and water stress: Role of osmolytes as enzyme protectant. Journal of Plant Physiology 162: 854-864.

3. Greenway H, Munns R (1980) Mechanisms of salt tolerance in nonhalophytes. Annual review of plant physiology 31: 149-190.

4. Alia, Saradhi PP, Mohanty P (1997) Involvement of proline in protecting thylakoid membranes against free radical-induced photodamage. Journal of Photochemistry and Photobiology B: Biology 38: 253-257.

5. Signorelli S, Coitiño EL, Borsani O, Monza J (2014) Molecular mechanisms for the reaction between •OH radicals and proline: Insights on the role as reactive oxygen species scavenger in plant stress. Journal of Physical Chemistry B 118: 37-47.

6. Matysik J, Alia, Bhalu B, Mohanty P (2002) Molecular mechanisms of quenching of reactive oxygen species by proline under stress in plants. Current Science 82: 525-532.

7. Hare PD, Cress WA (1997) Metabolic implications of stress-induced proline accumulation in plants. Plant Growth Regulation 21: 79-102.

8. Mallick N (2004) Copper-induced oxidative stress in the chlorophycean microalga Chlorella vulgaris: response of the antioxidant system. J Plant Physiol 161: 591-597.

9. Zhang LP, Mehta SK, Liu ZP, Yang ZM (2008) Copper-induced proline synthesis is associated with nitric oxide generation in Chlamydomonas reinhardtii. Plant and Cell Physiology 49: 411-419.

10. Mehta SK, Gaur JP (1999) Heavy metal-induced proline accumulation and its role in ameliorating metal toxicity in Chlorella vulgaris. New Phytologist 143: 253-259.

11. Siripornadulsil S, Traina S, Verma DPS, Sayre RT (2002) Molecular mechanisms of proline-mediated tolerance to toxic heavy metals in transgenic microalgae. Plant Cell 14: 2837-2847.

12. Rochaix JD, Merchant S (1998) The Molecular Biology of Chloroplasts and Mitochondria in Chlamydomonas: Springer.

13. Varshney RK, Koebner RMD (2010) Model Plants and Crop Improvement: Taylor & Francis.

14. Krell A, Funck D, Plettner I, John U, Dieckmann G (2007) Regulation of proline metabolism under salt stress in the psychrophilic diatom Fragilariopsis cylindrus (Bacillariophyceae). Journal of Phycology 43: 753-762.

15. Hong Z, Lakkineni K, Zhang Z, Verma DPS (2000) Removal of Feedback Inhibition of Δ(1)-Pyrroline-5-Carboxylate Synthetase Results in Increased Proline Accumulation and Protection of Plants from Osmotic Stress. Plant Physiology 122: 1129-1136.

16. Hu CA, Delauney AJ, Verma DP (1992) A bifunctional enzyme (delta 1-pyrroline-5-carboxylate synthetase) catalyzes the first two steps in proline biosynthesis in plants. Proceedings of the National Academy of Sciences of the United States of America 89: 9354-9358.

17. Merchant SS, Prochnik SE, Vallon O, Harris EH, Karpowicz SJ, et al. (2007) The Chlamydomonas genome reveals the evolution of key animal and plant functions. Science 318: 245-250.

18. Gamper H, Moses V (1974) Enzyme organization in the proline biosynthetic pathway of Escherichia coli. Biochimica et Biophysica Acta (BBA) - General Subjects 354: 75-87.

19. Abbott DA, Suir E, Duong G-H, de Hulster E, Pronk JT, et al. (2009) Catalase overexpression reduces lactic acid-induced oxidative stress in Saccharomyces cerevisiae. Applied and environmental microbiology 75: 2320-2325.

20. Xu B, Jahic M, Blomsten G, Enfors S-O (1999) Glucose overflow metabolism and mixed-acid fermentation in aerobic large-scale fed-batch processes with Escherichia coli. Applied microbiology and biotechnology 51: 564-571.

21. Lahtvee P-J, Kumar R, Hallström BM, Nielsen J (2016) Adaptation to different types of stress converge on mitochondrial metabolism. Molecular biology of the cell 27: 2505-2514.

22. Sakamoto A, Murata N (2002) The role of glycine betaine in the protection of plants from stress: clues from transgenic plants. Plant, Cell & Environment 25: 163-171.

23. Ashraf M, Foolad M (2007) Roles of glycine betaine and proline in improving plant abiotic stress resistance. Environmental and Experimental Botany 59: 206-216.

24. Rhodes D, Hanson AD (1993) Quaternary ammonium and tertiary sulfonium compounds in higher plants. Annual Review of Plant Physiology and Plant Molecular Biology 44: 357-384.

25. Nomura M, Ishitani M, Takabe T, Rai AK, Takabe T (1995) Synechococcus Sp Pcc7942 Transformed with Escherichia-Coli Bet Genes Produces Glycine Betaine from Choline and Acquires Resistance to Salt Stress. Plant Physiology 107: 703-708.

26. Gage DA, Rhodes D, Nolte KD, Hicks WA, Leustek T, et al. (1997) A new route for synthesis of dimethylsulphoniopropionate in marine algae. Nature 387: 891-894.

27. Hanson AD, Gage DA, Kiene R, Visscher P, Keller M, et al. (1996) 3-Dimethylsulfoniopionate Biosynthesis and use by Flowering Plants. Biological and Environmental Chemistry of DMSP and Related Sulfonium Compounds: Springer US. pp. 75-86.

28. Summers PS, Nolte KD, Cooper AJL, Borgeas H, Leustek T, et al. (1998) Identification and Stereospecificity of the First Three Enzymes of 3-Dimethylsulfoniopropionate Biosynthesis in a Chlorophyte Alga. Plant Physiology 116: 369-378.

29. Wolfe GV, Steinke M, Kirst GO (1997) Grazing-activated chemical defence in a unicellular marine alga. 387: 894-897.

30. Popova LG, Shumkova GA, Andreev IM, Balnokin YV (2005) Functional identification of electrogenic Na+-translocating ATPase in the plasma membrane of the halotolerant microalga Dunaliella maritima. FEBS Letters 579: 5002-5006.

31. Ruiz FA, Marchesini N, Seufferheld M, Govindjee, Docampo R (2001) The polyphosphate bodies of Chlamydomonas reinhardtii possess a proton-pumping pyrophosphatase and are similar to acidocalcisomes. J Biol Chem 276: 46196-46203.

32. Galinski EA, Truper HG (1994) Microbial Behavior in Salt-Stressed Ecosystems. Fems Microbiology Reviews 15: 95-108.

33. Williamson JD, Jennings DB, Guo WW, Pharr DM, Ehrenshaft M (2002) Sugar alcohols, salt stress, and fungal resistance: Polyols - Multifunctional plant protection? Journal of the American Society for Horticultural Science 127: 467-473.

34. Ginzburg M (1987) Dunaliella - a Green-Alga Adapted to Salt. Advances in Botanical Research Incorporating Advances in Plant Pathology 14: 93-183.

35. Borowitzka L, Brown A (1974) The salt relations of marine and halophilic species of the unicellular green alga,Dunaliella. Archives of Microbiology 96: 37-52.

36. Band CJ, Arredondo-Vega BO, Vazquez-Duhalt R, Greppin H (1992) Effect of a salt-osmotic upshock on the edaphic microalga Neochloris oleoabundans. Plant, Cell & Environment 15: 129-133.
