# Supplementary material for: Neochloris oleoabundans is worth its salt: Transcriptomic analysis under salt and nitrogen stress
Source: PLoS One. 2018 Apr 13;13(4):e0194834. doi: 10.1371/journal.pone.0194834 (PMC5898717; doi:10.1371/journal.pone.0194834)
Supplement: S2 Table — (DOCX) [file pone.0194834.s006.docx]

| **EC number** | **FPKM** | **FPKM fold change (log2)** | | |
| --- | --- | --- | --- | --- |
|  |  | **FN+ vs FN-** | **FN+ vs SN+** | **FN+ vs SN-** |
| **EC:1.1.99.1** | 310 | -1.1 | 0.1 | -2.3 |
| **EC:2.6.1.5** | 162 | 0.0 | 0.5 | 0.1 |
| **EC:2.6.1.57** | 77 | -0.1 | 0.9 | 0.6 |
| **EC:2.4.1.1** | 38 | 0.6 | 0.8 | 1.4 |
| **EC:3.2.1.1** | 279 | 0.3 | 0.4 | 0.3 |
| **EC:3.1.1.3** | 1734 | -0.3 | -0.9 | -0.6 |
