# Supplementary material for: Neochloris oleoabundans is worth its salt: Transcriptomic analysis under salt and nitrogen stress
Source: PLoS One. 2018 Apr 13;13(4):e0194834. doi: 10.1371/journal.pone.0194834 (PMC5898717; doi:10.1371/journal.pone.0194834)
Supplement: S3 Table — (DOCX) [file pone.0194834.s007.docx]

| ***contigID*** | ***FN+*** | ***FN-*** | ***SN+*** | ***SN-*** | ***EC number*** | ***FPKM FN+*** | ***log2 FN+/FN-*** | ***log2 FN+/SN+*** | ***log2 FN+/SN-*** |
| --- | --- | --- | --- | --- | --- | --- | --- | --- | --- |
| contig-100_7308:541-1398 | 2 | 5 | 538 | 378 | **EC:3.6.1.3** | 2 | 1.6 | 8.3 | 7.8 |
| contig-100_5520:17-607 | 2 | 7 | 715 | 612 | **EC:3.6.1.3** | 2 | 1.5 | 8.3 | 8.0 |
| contig-100_2762:95-1692 | 5 | 9 | 71 | 180 | **EC:3.6.1.3** | 5 | 1.0 | 3.9 | 5.2 |
| contig-100_5924:1287-1748 | 34 | 43 | 240 | 247 | **EC:3.6.1.3** | 34 | 0.4 | 2.8 | 2.9 |
| contig-100_186:4802-6362 | 17 | 25 | 119 | 156 | **EC:3.6.1.3** | 17 | 0.5 | 2.8 | 3.2 |
| contig-100_9352:5-571 | 4 | 9 | 25 | 31 | **EC:3.6.1.3** | 4 | 1.1 | 2.6 | 2.9 |
| contig-100_150:2822-4086 | 47 | 107 | 256 | 321 | **EC:3.6.1.3** | 47 | 1.2 | 2.4 | 2.8 |
| contig-100_7483:65-1414 | 90 | 134 | 477 | 310 | **EC:3.6.1.3** | 90 | 0.6 | 2.4 | 1.8 |
| contig-100_3981:656-1456 | 26 | 27 | 134 | 131 | **EC:3.6.1.3** | 26 | 0.1 | 2.4 | 2.4 |
| contig-100_150:1714-2391 | 23 | 44 | 119 | 134 | **EC:3.6.1.3** | 23 | 0.9 | 2.4 | 2.5 |
| contig-100_150:607-1266 | 39 | 68 | 197 | 195 | **EC:3.6.1.3** | 39 | 0.8 | 2.3 | 2.3 |
| contig-100_9108:472-1068 | 57 | 58 | 244 | 150 | **EC:3.6.1.3** | 57 | 0.0 | 2.1 | 1.4 |
| contig-100_11187:85-846 | 20 | 56 | 80 | 347 | **EC:3.6.1.3** | 20 | 1.5 | 2.0 | 4.1 |
| contig-100_309:3647-5893 | 51 | 78 | 203 | 297 | **EC:3.6.1.3** | 51 | 0.6 | 2.0 | 2.5 |
| contig-100_8783:531-1187 | 27 | 40 | 93 | 89 | **EC:3.6.1.3** | 27 | 0.6 | 1.8 | 1.7 |
| contig-100_8508:179-1072 | 8 | 7 | 27 | 27 | **EC:3.6.1.3** | 8 | -0.2 | 1.8 | 1.8 |
| contig-100_9200:503-901 | 19 | 50 | 63 | 89 | **EC:3.6.1.3** | 19 | 1.4 | 1.8 | 2.3 |
| contig-100_1730:7-3315 | 21 | 29 | 68 | 76 | **EC:3.6.1.3** | 21 | 0.5 | 1.7 | 1.9 |
| contig-100_3665:419-2454 | 21 | 19 | 65 | 101 | **EC:3.6.1.3** | 21 | -0.1 | 1.6 | 2.3 |
| contig-100_10561:25-638 | 15 | 12 | 44 | 43 | **EC:3.6.1.3** | 15 | -0.3 | 1.6 | 1.5 |
| contig-100_1811:2418-2939 | 14 | 26 | 36 | 43 | **EC:3.6.1.3** | 14 | 0.9 | 1.4 | 1.7 |
| contig-100_2472:1072-1512 | 34 | 50 | 89 | 138 | **EC:3.6.1.3** | 34 | 0.5 | 1.4 | 2.0 |
| contig-100_213:290-1246 | 36 | 117 | 77 | 160 | **EC:3.6.1.3** | 36 | 1.7 | 1.1 | 2.2 |
| contig-100_10425:32-811 | 38 | 10 | 70 | 1 | **EC:3.6.1.3** | 38 | -2.0 | 0.9 | -5.3 |
| contig-100_7158:22-1418 | 39 | 6 | 52 | 2 | **EC:3.6.1.3** | 39 | -2.7 | 0.4 | -4.2 |
| contig-100_948:79-2019 | 54 | 8 | 72 | 1 | **EC:3.6.1.3** | 54 | -2.7 | 0.4 | -5.3 |
| contig-100_5308:213-1888 | 16 | 8 | 20 | 2 | **EC:3.6.1.3** | 16 | -1.1 | 0.3 | -3.3 |
| contig-100_10292:20-850 | 493 | 152 | 578 | 538 | **EC:3.6.1.3** | 493 | -1.7 | 0.2 | 0.1 |
| contig-100_2690:756-1310 | 17 | 2 | 19 | 1 | **EC:3.6.1.3** | 17 | -2.8 | 0.2 | -5.0 |
| contig-100_8852:713-1180 | 397 | 129 | 412 | 441 | **EC:3.6.1.3** | 397 | -1.6 | 0.1 | 0.2 |
| contig-100_542:142-5340 | 12 | 1 | 10 | 2 | **EC:3.6.1.3** | 12 | -3.1 | -0.2 | -2.7 |
| contig-100_2690:9-632 | 48 | 8 | 41 | 1 | **EC:3.6.1.3** | 48 | -2.5 | -0.2 | -6.1 |
| contig-100_2690:1547-2845 | 40 | 5 | 31 | 0 | **EC:3.6.1.3** | 40 | -3.0 | -0.4 | -9.2 |
| contig-100_514:3782-5335 | 24 | 9 | 18 | 6 | **EC:3.6.1.3** | 24 | -1.3 | -0.4 | -2.1 |
| contig-100_12078:270-785 | 1 | 1 | 0 | 4 | **EC:3.6.1.3** | 1 | -0.3 | -0.6 | 2.6 |
| contig-100_17017:44-501 | 85 | 35 | 53 | 29 | **EC:3.6.1.3** | 85 | -1.3 | -0.7 | -1.6 |
| contig-100_550:296-1897 | 6 | 17 | 2 | 2 | **EC:3.6.1.3** | 6 | 1.6 | -1.3 | -1.4 |
| contig-100_11812:63-807 | 137 | 114 | 47 | 76 | **EC:3.6.1.3** | 137 | -0.3 | -1.5 | -0.9 |
| contig-100_16157:100-563 | 234 | 138 | 78 | 133 | **EC:3.6.1.3** | 234 | -0.8 | -1.6 | -0.8 |
| contig-100_3864:255-2380 | 81 | 20 | 27 | 4 | **EC:3.6.1.3** | 81 | -2.0 | -1.6 | -4.3 |
| contig-100_11959:7-786 | 305 | 173 | 92 | 188 | **EC:3.6.1.3** | 305 | -0.8 | -1.7 | -0.7 |
| contig-100_15630:37-584 | 153 | 83 | 44 | 64 | **EC:3.6.1.3** | 153 | -0.9 | -1.8 | -1.3 |
| contig-100_13619:48-676 | 71 | 31 | 18 | 20 | **EC:3.6.1.3** | 71 | -1.2 | -2.0 | -1.8 |
| contig-100_13535:297-695 | 269 | 314 | 64 | 139 | **EC:3.6.1.3** | 269 | 0.2 | -2.1 | -1.0 |
| contig-100_11874:38-829 | 60 | 18 | 13 | 12 | **EC:3.6.1.3** | 60 | -1.7 | -2.2 | -2.3 |
| contig-100_6:24-13086 | 67 | 19 | 11 | 13 | **EC:3.6.1.3** | 67 | -1.8 | -2.6 | -2.3 |
| contig-100_11049:29-418 | 57 | 19 | 8 | 12 | **EC:3.6.1.3** | 57 | -1.6 | -2.8 | -2.2 |
| contig-100_2386:2609-3091 | 57 | 79 | 6 | 28 | **EC:3.6.1.3** | 57 | 0.5 | -3.3 | -1.0 |
| contig-100_662:212-5114 | 331 | 129 | 31 | 40 | **EC:3.6.1.3** | 331 | -1.4 | -3.4 | -3.1 |
| contig-100_589:152-643 | 1089 | 553 | 89 | 170 | **EC:3.6.1.3** | 1089 | -1.0 | -3.6 | -2.7 |
| contig-100_2693:97-2651 | 863 | 401 | 65 | 121 | **EC:3.6.1.3** | 863 | -1.1 | -3.7 | -2.8 |
| contig-100_3741:301-2334 | 202 | 47 | 14 | 22 | **EC:3.6.1.3** | 202 | -2.1 | -3.8 | -3.2 |
| contig-100_112:5203-7668 | 212 | 450 | 560 | 863 | **EC:3.6.3.9** | 212 | 1.1 | 1.4 | 2.0 |
| contig-100_10686:3-683 | 66 | 160 | 212 | 310 | **EC:3.6.3.6** | 66 | 1.3 | 1.7 | 2.2 |
| contig-100_8061:1-483 | 114 | 268 | 339 | 493 | **EC:3.6.3.6** | 114 | 1.2 | 1.6 | 2.1 |
| contig-100_3090:1255-2283 | 0 | 1 | 7 | 1 | **EC:3.6.3.2** | 0 | 9.7 | 12.8 | 10.1 |
